# Checklist of thallus-forming Laboulbeniomycetes from Belgium and the Netherlands, including *Hesperomyces halyziae* and *Laboulbenia quarantenae* spp. nov.

**DOI:** 10.3897/mycokeys.71.53421

**Published:** 2020-07-30

**Authors:** Danny Haelewaters, André De Kesel

**Affiliations:** 1 Purdue University, West Lafayette, Indiana, United States of America Purdue University West Lafayette United States of America; 2 University of South Bohemia, České Budějovice, Czech Republic University of South Bohemia Budějovice Czech Republic; 3 Ghent University, Ghent, Belgium Ghent University Ghent Belgium; 4 Meise Botanic Garden, Meise, Belgium Meise Botanic Garden Meise Belgium

**Keywords:** 2 new taxa, arthropod-associated fungi, Ascomycota, Herpomycetales, integrative taxonomy, key, Laboulbeniales

## Abstract

In this paper we present an updated checklist of thallus-forming Laboulbeniomycetes (Ascomycota, Pezizomycotina), that is, the orders Herpomycetales and Laboulbeniales, from Belgium and the Netherlands. Two species are newly described based on morphology, molecular data (ITS, LSU ribosomal DNA) and ecology (host association). These are *Hesperomyces halyziae* on *Halyzia sedecimguttata* (Coleoptera, Coccinellidae) from both countries and *Laboulbenia quarantenae* on *Bembidion biguttatum* (Coleoptera, Carabidae) from Belgium. In addition, nine new country records are presented. For Belgium: *Laboulbenia aubryi* on *Amara aranea* (Coleoptera, Carabidae) and *Rhachomyces spinosus* on *Syntomus foveatus* (Coleoptera, Carabidae). For the Netherlands: *Chitonomyces melanurus* on *Laccophilus minutus* (Coleoptera, Dytiscidae), *Euphoriomyces agathidii* on *Agathidium laevigatum* (Coleoptera, Leiodidae), *Laboulbenia fasciculata* on *Omophron limbatum* (Coleoptera, Carabidae), *Laboulbenia metableti* on *Syntomus foveatus* and *S. truncatellus* (Coleoptera, Carabidae), *Laboulbenia pseudomasei* on *Pterostichus melanarius* (Coleoptera, Carabidae), *Rhachomyces canariensis* on *Trechus obtusus* (Coleoptera, Carabidae), and *Stigmatomyces hydrelliae* on *Hydrellia albilabris* (Diptera, Ephydridae). Finally, an identification key to 140 species of thallus-forming Laboulbeniomycetes in Belgium and the Netherlands is provided. Based on the combined data, we are able to identify mutual gaps that need to be filled as well as weigh the impact of chosen strategies (fieldwork, museum collections) and techniques in these neighboring countries. The aim of this work is to serve as a reference for studying Laboulbeniomycetes fungi in Europe.

## Introduction

Herpomycetales and Laboulbeniales are two orders within the class Laboulbeniomycetes (Ascomycota, Pezizomycotina), consisting of arthropod-associated biotrophs. Both orders are unique among related fungi in that they do not form hyphae; instead, *thalli* are produced by mitotic divisions from a two-celled ascospore. Herpomycetales was recently described and includes a single genus, *Herpomyces* Thaxt., with 27 described species–all associated with cockroaches (Blattodea) (Haelewaters et al. 2019b; Gutierrez et al. 2020). The Laboulbeniales order, on the other hand, successfully radiated on a wide range of hosts. Representatives of this order can be found in three arthropod subphyla, including mites and harvestmen (in subphylum Chelicerata), millipedes (in subphylum Myriapoda), and many orders of true insects (in subphylum Hexapoda). The vast majority of about 2,325 described species (Kirk 2019) are known from beetles (order Coleoptera), hence the common name once introduced for the group, “beetle hangers” (Cooke 1892). The early taxonomic history of these fungi is fraught with confusion (Blackwell et al. 2020), but the incorporation of sequence data has led to a conclusive placement of these fungi within Ascomycota (Blackwell 1994; Weir and Blackwell 2001; Schoch et al. 2009).

Early studies on Laboulbeniales (including *Herpomyces* at that time) in Belgium and the Netherlands are scarce. In Belgium, Collart (1945, 1947) and Rammeloo (1986) made noteworthy contributions, followed by multiple publications by De Kesel and colleagues (1989–present). The Laboulbeniomycetes from Belgium were for the first time summarized by De Kesel and Rammeloo (1992), who reported 1 species of *Herpomyces* and 47 species of Laboulbeniales. De Kesel et al. (2020) provided an updated – and illustrated – *Catalogue of the Laboulbeniomycetes of Belgium*, with a total of 115 species (3 Herpomycetales, 112 Laboulbeniales) from 222 host species. For more details regarding the study of Herpomycetales and Laboulbeniales in Belgium, we refer to De Kesel and Rammeloo (1992) and De Kesel et al. (2020). In the Netherlands, thus far, no effort has been made to publish a checklist.

The study of Laboulbeniales in the Netherlands started during a meeting of the Dutch Entomological Society in 1906, triggered by a question from Dr. Johannes P. Lotsy, then director of the “Rijksherbarium” (Leiden). In response, Prof. Dr. De Meijere remembered that he once observed an infected *Drosophila funebris* (Fabricius, 1787) fly, collected at the ARTIS Amsterdam Royal Zoo in 1904, but had not thought it worthy of mention at the time. Recent infected material of *D. funebris* from nature reserve De Kaaistoep has thus far always been associated with *Stigmatomyces entomophilus* (Peck) Thaxt. (Haelewaters et al. 2015b) and hence it is likely that *S. entomophilus* represents the very first report of Laboulbeniales from the Netherlands. The first published account was a developmental study of *Stigmatomyces baeri* H. Karst. by Boedijn (1923). The fungus was found on an atypical host – *Fannia canicularis* (Linnaeus, 1761); this fly is the only reported host for *Fanniomyces ceratophorus* (Whisler) T. Majewski, which is morphologically different from Boedijn’s (1923) drawings. We agree with Thaxter (1931) that the fungus was probably correctly identified by Boedijn, but perhaps the host was not.

Next, in the 1930s, only two species of Laboulbeniales were reported in the Netherlands: *Laboulbenia cristata* Thaxt. from *Paederus riparius* (Linnaeus, 1758) (Kossen 1936, 1938) and *Laboulbenia flagellata* Peyr. from *Platynus* spp. (Zaneveld 1938). It was not until Abraham Middelhoek (1906–1968) that the number of reported species of Laboulbeniales in the Netherlands would increase by 25 (Middelhoek 1941, 1942, 1943a, b, c, d, 1945, 1947a, b, 1949). Middelhoek was first an artist who, among other things, made stained glass windows. Only after World War II, he studied biology and raised an interest in fungi, particularly the Laboulbeniales. After Middelhoek, Laboulbeniales were forgotten about in the Netherlands except for a single paper by Meijer (1975), who proposed to use Laboulbeniales fungi as “biological tags” to trace migration patterns. Since 2012, Haelewaters and colleagues have published several papers dealing with Laboulbeniales in the Netherlands, which together have more than doubled the number of reported species in this country (De Kesel and Gerstmans 2012; Haelewaters 2012, 2013; Haelewaters et al. 2012a, b, 2014, 2015a, b, 2020; De Kesel et al. 2013; Haelewaters and De Kesel 2013; De Kesel and Haelewaters 2014, 2019; Haelewaters and van Wielink 2016). To date, 79 species of Laboulbeniales are reported from the Netherlands.

In this contribution we compile all available data from Belgium and the Netherlands. Keeping in mind that both countries show some geographical differences, especially due to specific soils and increasing altitude in the southern part of Belgium, we think a combined checklist makes sense at this point. This is mainly because the sampling effort for Laboulbeniomycetes in the southern part of Belgium has been much lower compared to the northern and central areas of the country (De Kesel et al. 2020). As a result, the bulk of Belgian and Dutch records come from biogeographically comparable regions. The here presented checklist is useful to illustrate where mutual gaps need to be filled and what the impact has been of the chosen strategies (fieldwork, museum collections) and trapping techniques. In combination with the recently published Belgian catalogue (De Kesel et al. 2020) presenting illustrations and identification keys to 115 taxa, this checklist will serve as a reference for mycologists, students, and scholars studying Laboulbeniomycetes fungi. In addition, this work is an appropriate starting point for an updated checklist of thallus-forming Laboulbeniomycetes from Europe–an ongoing project that needs to be updated, three decades after the massive undertaking of Santamaría et al. (1991).

## Materials and methods

### Specimen collection and morphological study

Insects were collected in Belgium and the Netherlands using pitfall traps and on an illuminated white screen at night. Specimens were preserved in 96–99% ethanol until they were screened for presence of thalli of Laboulbeniomycetes at 20–50× magnification. Thalli were removed from the host at the foot and mounted in Amann solution following the methods in De Kesel et al. (2020). Drawings and measurements were made using a BX51 light microscope (Olympus, Tokyo, Japan) with drawing tube, digital camera, and AnalySIS software (Soft Imaging System GmbH, Münster, Germany); or an an Olympus BH2 bright field compound microscope with SC30 camera and cellSens 1.18 imaging software.

Infected hosts found in Belgium and the Netherlands are preserved at Meise Botanic Garden (BR) and the Brabant Museum of Nature, Tilburg (NNKN), respectively. Microscope slides of Laboulbeniales are deposited at BR, FH, GENT, and NMBT (Thiers continuously updated).

### DNA extraction, PCR amplification, sequencing

Three thalli of *Laboulbenia quarantenae* sp. nov. were used for DNA isolation using the REPLI-g Single Cell Kit (Qiagen, Stanford, California) with modifications (Haelewaters et al. 2019b). The DNA extract was stored at -20 °C until PCR amplification. Recent studies found that even though the internal transcribed spacer (ITS) region is a good marker for species delimitation in Laboulbeniomycetes, it is difficult to amplify in this group. Instead, the large subunit (LSU) of the ribosomal RNA gene has been put forward as a secondary barcode because it is easy to amplify and provides high discriminative resolution at species-level (e.g., Haelewaters et al. 2018; Sundberg et al. 2018b; Walker et al. 2018; Liu et al. 2020). The partial LSU was amplified using primers LIC15R (Miadlikowska et al. 2002) and LR6 (Vilgalys and Hester 1990). Sequencing was outsourced to Macrogen Europe (Amsterdam, the Netherlands) with the same PCR primers and an additional reverse primer, LR3 (Vilgalys and Hester 1990). Resulting forward and both reverse sequence reads were assembled and edited with Sequencher version 5.2.3 (Gene Codes Corporation, Ann Arbor, Michigan).

For *Hesperomyces halyziae*, molecular work had been done previously (Haelewaters et al. 2018). DNA was extracted using the Extract-N-Amp Plant PCR Kit (Sigma-Aldrich, St. Louis, Missouri) (methods in Haelewaters et al. 2015c). Seven thalli were placed in a 1.5 mL tube with 40 µL of Extraction Solution and sterilized sand. The tube was then placed in a FastPrep FP120 Cell Disrupter (Thermo Fisher Scientific, Waltham, Massachusetts) to mechanically crush fungal material at 5.5 m/s for 20 sec, and then on a heating block to incubate at 95 °C for 10 min. Finally, a total of 120 µL Dilution Solution was added to the mixture. Because we needed to define “*H. virescens* sensu stricto”, additional extractions from single *Hesperomyces* thalli removed from *Chilocorus stigma* (Say, 1835) were performed using the REPLI-g Single Cell Kit with modifications. Amplification of the ITS was done using primers ITS1f (Gardes and Bruns 1993) and ITS4 (White et al. 1990) as well as *Hesperomyces*-specific primers ITShespL and ITShespR (Haelewaters et al. 2019b). Purification and sequencing (same primers) of these PCR products were outsourced to Genewiz (Plainfield, New Jersey).

### Phylogenetic analyses

Methods for both datasets – ITS for *Hesperomyces*, LSU for *Laboulbenia* – were largely identical. Sequences were downloaded from NCBI GenBank (https://www.ncbi.nlm.nih.gov/genbank/) and supplemented with sequences that were generated during this study. Sequences were aligned using MUSCLE version 3.7 (Edgar 2004), which is available on the CIPRES Science Gateway V. 3.3 (Miller et al. 2010). After alignment of the ITS dataset, partial SSU and partial LSU were removed by looking for the motifs 5’-ATCATTA-3’ (3’ end of SSU) and 5’-TGACCT-3’ (5’ start of LSU), and deleting downstream and upstream sequence data, respectively (Baral et al. 2018). For the LSU dataset, we unsuccessfully searched for the 5’-TGACCT-3’ motif. We then looked for the motif following 5’-TGACCT-3’ in a *Hesperomyces* sequence (GenBank acc. no. MG757513), which is 5’-CGGAT-3’, found this motif in the *Laboulbenia* dataset, and then realized that the 5’ start of LSU in *Laboulbenia* includes one nucleotide substitution compared to the conventional motif: 5’-TGGCCT-3’. We deleted the downstream sequence data to remove partial ITS. Next, ambiguously aligned regions and uninformative positions were removed using the command line version of trimAl v1.2 (Capella-Gutiérrez et al. 2009) with gap threshold = 0.6 and minimal coverage = 0.5. Models of nucleotide substitution were selected by considering the Akaike Information Criterion corrected for small samples (AICc) with ModelFinder Plus (Kalyaanamoorthy et al. 2017). Maximum likelihood (ML) was inferred for each dataset under the selected model with IQ-TREE (Nguyen et al. 2015; Chernomor et al. 2016). Ultrafast bootstrap (BS) analysis with 1000 replicates estimated branch support in the ML trees (Hoang et al. 2018).

Bayesian analyses were done using a Markov chain Monte Carlo (MCMC) coalescent approach implemented in BEAST 1.8.4 (Drummond et al. 2012), with a strict clock assuming a constant rate of evolution across the tree, a Yule Speciation tree prior (Yule 1925; Gernhard 2008), and the nucleotide substitution model as selected by jModelTest 2.1 (Darriba et al. 2012) under the AICc criterion. For each dataset, four runs were performed from a random starting tree for 10 million generations with a sampling frequency of 1000. All settings were entered in BEAUti 1.8.4 to generate an XML file, which was run in BEAST on the CIPRES Science Gateway (Miller et al. 2010). Resulting log files were entered in Tracer version 1.6 (Rambaut et al. 2014) to check MCMC trace plots for convergence and to assess effective sample sizes (ESS). A standard 10% burn-in was used resulting in overall ESS values of well above 200 for all sampled parameters. After removal of 10% burn-in, trees files were combined in LogCombiner 1.8.4. TreeAnnotator 1.8.4 was used to generate consensus trees with 0% burn-in and to infer the Maximum Clade Credibility tree with highest product of individual clade posterior probabilities (pp) for both datasets.

Trees with ML BS and Bayesian pp were visualized in FigTree version 1.4.3 (http://tree.bio.ed.ac.uk/software/figtree/) and edited in Adobe Illustrator 2020 version 24.1.1 (San Jose, California).

### Checklist

For the checklist of thallus-forming Laboulbeniomycetes from Belgium and the Netherlands, we used De Kesel et al. (2020) for Belgium and all available published papers (since 1938 up to 2020) for the Netherlands. Laboulbeniomycetes and their hosts are listed alphabetically, starting with Herpomycetales, followed by Laboulbeniales. Fungal species are numbered throughout (1–140), authority and reference to the protologue are presented. For each fungus, hosts are presented alphabetically, with classification (order, family) and country in which the association has been reported: “Be” for Belgium, “Nl” for the Netherlands. No detailed collection information is shown except for new country records. In several instances, taxonomic notes are provided. Hosts are according to Vorst (2010) and Beccaloni et al. (2014). Names of fungi correspond to Index Fungorum (2020).

### Identification key

The key to species of Laboulbeniomycetes in Belgium and the Netherlands is based on diagnostic characters referring to morphology and/or host taxa. It requires microscope equipment and morphological study as described in Benjamin (1971), Huldén (1983), Majewski (1994), Santamaría (1998), and De Kesel et al. (2020). Terminology follows Tavares (1985), Santamaría (1998, 2003), and De Kesel et al. (2020).

## Results

**Figure 1.**
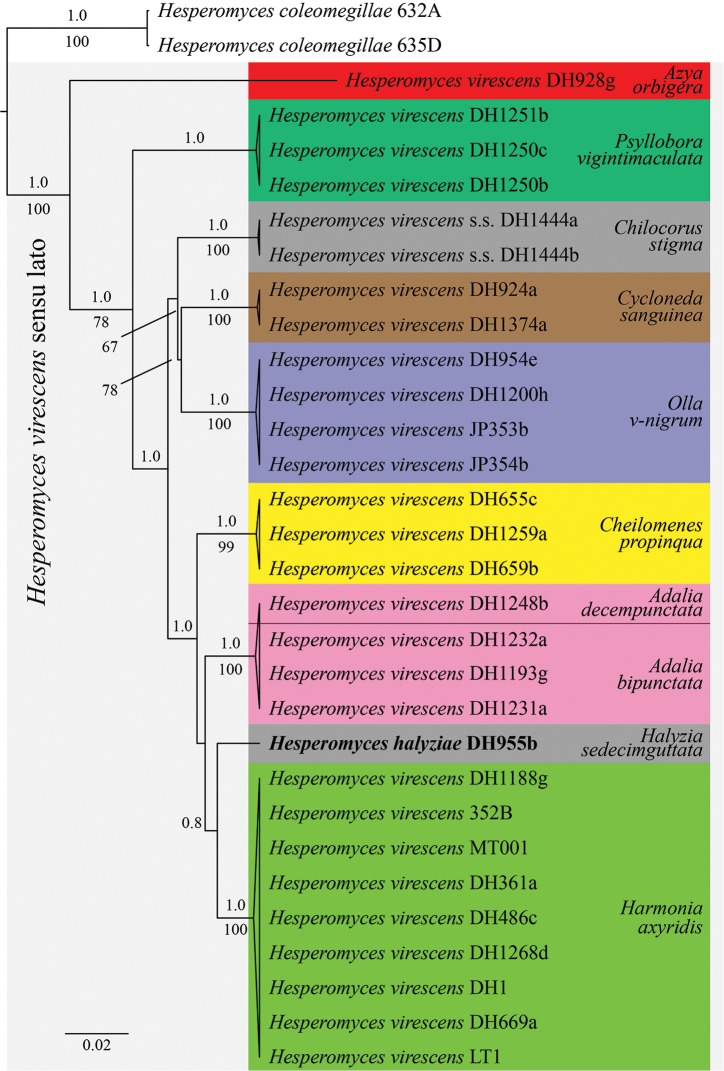
Maximum clade creditability tree of *Hesperomyces* isolates reconstructed from an ITS dataset, with *H. coleomegillae* as outgroup. The topology is the result of Bayesian inference performed with BEAST. For each node, ML BS (≥ 65) and Bayesian pp (≥ 0.7) are presented above/below the branch leading to that node. *Hesperomyces virescens* sensu lato is highlighted with light gray shading, isolates are color-coded by host; *H. virescens* sensu stricto and *H. halyziae* sp. nov. are highlighted with dark gray shading.

The ITS dataset consisted of 31 *Hesperomyces* sequences (Table 1) and 724 characters, of which 462 were constant and 198 were parsimony-informative. The selected nucleotide substitution model under AICc was TVM+F+G4 (-lnL = 2790.545, ModelFinder Plus) and TVM+G (-lnL = 2786.8769, jModelTest 2). The *Hesperomyces virescens* sensu lato (Haelewaters et al. 2018) clade has maximum support from both ML and Bayesian analyses (Figure 1). Each of the nine clades within *H. virescens* s.l. consists of isolates from thalli removed from a single host species, except for the *Adalia* clade, which includes isolates from both *A. bipunctata* and *A. decempunctata*. One of the clades consists of isolates from *Chilocorus stigma*, the host on which *H. virescens* was originally described (Thaxter 1891). This clade, representative of *Hesperomyces virescens* sensu stricto, receives maximum support. The single isolate of *Hesperomyces halyziae*, from *Halyzia sedecimguttata*, is placed as sister to *H. virescens* s.l. from *Harmonia axyridis* (Pallas, 1773) (pp = 0.8).

**Table 1.** *Hesperomyces* sequences used in phylogenetic analysis of the ITS dataset. Asterisks (*) indicate sequences that were generated during the course of this study.

Species	Host	Isolate	GenBank (ITS)	Reference
*Hesperomyces coleomegillae*	*Coleomegilla maculata*	632A	KF192888	Goldmann et al. (2013)
	*Coleomegilla maculate*	635D	KF192906	Goldmann et al. (2013)
***Hesperomyces halyziae***	*Halyzia sedecimguttata*	D. Haelew. 955b	MG757813	Haelewaters et al. (2018)
*Hesperomyces virescens* s.s.	*Chilocorus stigma*	D. Haelew. 1444a	MT373697*	This paper
	*Chilocorus stigma*	D. Haelew. 1444b	MT373698*	This paper
*Hesperomyces virescens* s.l.	*Adalia bipunctata*	D. Haelew. 1193g	MG757817	Haelewaters et al. (2018)
	*Adalia bipunctata*	D. Haelew. 1231a	MG757821	Haelewaters et al. (2018)
	*Adalia bipunctata*	D. Haelew. 1232a	MG757822	Haelewaters et al. (2018)
	*Adalia decempunctata*	D. Haelew. 1248b	MG757823	Haelewaters et al. (2018)
	*Azya orbigera*	D. Haelew. 928g	MG745343	Haelewaters et al. (2018)
	*Cheilomenes propinqua*	D. Haelew. 655c	MG757804	Haelewaters et al. (2018)
	*Cheilomenes propinqua*	D. Haelew. 659b	MG757805	Haelewaters et al. (2018)
	*Cheilomenes propinqua*	D. Haelew. 1259a	MG757828	Haelewaters et al. (2018)
	*Cycloneda sanguinea*	D. Haelew. 924a	MG757808	Haelewaters et al. (2018)
	*Cycloneda sanguinea*	D. Haelew. 1374a	MG757831	Haelewaters et al. (2018)
	*Harmonia axyridis*	352B	KF192916	Goldmann et al. (2013)
	*Harmonia axyridis*	D. Haelew. 361a	MG757801	Haelewaters et al. (2018)
	*Harmonia axyridis*	D. Haelew. 486c	KT800044	Haelewaters et al. (2015c)
	*Harmonia axyridis*	D. Haelew. 669a	MG757807	Haelewaters et al. (2018)
	*Harmonia axyridis*	D. Haelew. 1188g	MG438317	Haelewaters et al. (2019b)
	*Harmonia axyridis*	D. Haelew. 1268d	MG757830	Haelewaters et al. (2018)
	*Harmonia axyridis*	DH1	KF192920	Goldmann et al. (2013)
	*Harmonia axyridis*	LT1	KF192910	Goldmann et al. (2013)
	*Harmonia axyridis*	MT001	KT800048	Haelewaters et al. (2015c)
	*Olla v-nigrum*	D. Haelew. 954e	MG757812	Haelewaters et al. (2018)
	*Olla v-nigrum*	D. Haelew. 1200h	MG757819	Haelewaters et al. (2018)
	*Olla v-nigrum*	JP353b	MG757799	Haelewaters et al. (2018)
	*Olla v-nigrum*	JP354b	MG757800	Haelewaters et al. (2018)
	*Psyllobora vigintimaculata*	D. Haelew. 1250b	MG757825	Haelewaters et al. (2018)
	*Psyllobora vigintimaculata*	D. Haelew. 1250c	MG757826	Haelewaters et al. (2018)
	*Psyllobora vigintimaculata*	D. Haelew. 1251b	MG757827	Haelewaters et al. (2018)

The LSU dataset consisted of 24 *Laboulbenia* sequences (Table 2) and 682 characters, of which 558 were constant and 63 were parsimony-informative. The selected nucleotide substitution model under AICc was TN+F+G4 (-lnL = 1876.681, ModelFinder Plus) and TrN+G (-lnL = 1872.4616, jModelTest 2). Our phylogenetic analyses show nine distinct species, which are all supported. The relationships among species are unresolved in different places, but this is not unsurprising because of extremely limited taxon sampling. *Laboulbenia quarantenae* holds an unresolved position in the tree but is clearly separated from both *L. flagellata* and the morphologically similar *L. vulgaris*, confirming its status as a separate species. *Laboulbenia vulgaris* isolates E10T2 and E11T6, which originated from *Bembidion tetracolum*, are placed among isolates of the same species removed from *Ocys harpaloides*. Interestingly, and in accordance with De Weggheleire (2019) and Haelewaters et al. (2019a), *L. flagellata* falls apart in three species. However, only ten isolates are included, originating from six host species, none of which were reported in the protologue (Peyritsch 1873). As a result, it is too early to make taxonomic decisions *within* this problematic taxon.

**Table 2.** *Laboulbenia* sequences used in phylogenetic analysis of the LSU dataset. Asterisks (*) indicate sequences that were generated during the course of this study.

Species	Host	Isolate	GenBank (LSU)	Reference
*Laboulbenia bruchii*	*Neolema adunata*	D. Haelew. 1346b	MN394843	Haelewaters et al. (2019a)
*Laboulbenia collae*	*Paranchus albipes*	D. Haelew. 1456a	MN394844	Haelewaters et al. (2019a)
	*Paranchus albipes*	D. Haelew. 1456b	MN394845	Haelewaters et al. (2019a)
	*Paranchus albipes*	D. Haelew. 1461b	MN397131	Haelewaters et al. (2019a)
***Laboulbenia quarantenae***	*Bembidion biguttatum*, ADK6448	E13T12	MT371368*	This paper
*Laboulbenia flagellata*	*Agonum emarginatum*, ADK6428	E13T1	MT703825*	This paper
	*Agonum micans*, ADK6332	D. Haelew. 1457a	MN394851	Haelewaters et al. (2019a)
	*Agonum micans*, ADK6332	D. Haelew. 1457b	MN394852	Haelewaters et al. (2019a)
	*Agonum micans*, ADK6332	D. Haelew. 1457c	MN394853	Haelewaters et al. (2019a)
	*Agonum nigrum*, ADK6445	E13T11	MT703826*	This paper
	*Limodromus assimilis*, ADK6329-1	D. Haelew. 1454a	MN394849	Haelewaters et al. (2019a)
	*Limodromus assimilis*, ADK6329-1	D. Haelew. 1454b	MN394850	Haelewaters et al. (2019a)
	*Limodromus assimilis*, ADK6329-2	D. Haelew. 1458a	MN394854	Haelewaters et al. (2019a)
	*Loricera pilicornis*	H85-1	KY350538	Sundberg et al. (2018a)
	*Oxypselaphus obscurus*, ADK6374	E11T11	MT703824*	This paper
*Laboulbenia pedicellata*	*Dyschirius globosus*	H84-1	KY350537	Sundberg et al. (2018a)
*Laboulbenia systenae*	*Disonycha procera*	D. Haelew. 1342b	MN394858	Haelewaters et al. (2019a)
*Laboulbenia vulgaris*	*Bembidion tetracolum*, ADK6420	E10T2	MT703822*	This paper
	*Bembidion tetracolum*, ADK5557	E11E6	MT703823*	This paper
	*Ocys harpaloides*, ADK6330-1	D. Haelew. 1455a	MN397135	Haelewaters et al. (2019a)
	*Ocys harpaloides*, ADK6330-1	D. Haelew. 1455b	MN397136	Haelewaters et al. (2019a)
	*Ocys harpaloides*, ADK6330-2	D. Haelew. 1459a	MN397137	Haelewaters et al. (2019a)
	*Ocys harpaloides*, ADK6330-3	D. Haelew. 1460a	MN397138	Haelewaters et al. (2019a)
	*Ocys harpaloides*, ADK6353-1	E0T6	MT703821*	This paper

### Taxonomy

#### Hesperomyces halyziae

Taxon classificationFungiLaboulbenialesLaboulbeniaceae

Haelew. & De Kesel sp. nov.

71A06F79-F238-5F25-9E2C-3FDFDBCA0A74

835489

Figure 3

##### Etymology.

Referring to the host genus, *Halyzia*.

##### Diagnosis.

Morphologically very similar to other taxa within *H. virescens* sensu lato, but forming a distinct species supported by ITS data. The ITS sequence shares 95.8–97.9% identity with *H. virescens* s.l. from *Harmonia axyridis*, and 96.5–95.4% with *H. virescens* s.l. from *Adalia bipunctata*/*A. decempunctata*. Unique molecular synapomorphies in the ITS at positions 478, 517, 652.

##### Types.

***Holotype***: The Netherlands, Noord Brabant Province, Tilburg, nature reserve De Kaaistoep, 51.5333333N, 5.0166667E, 11 Aug. 2015, *leg.* H. Spijkers & P. van Wielink, on female *Halyzia sedecimguttata* (Linnaeus, 1758) (Coleoptera, Coccinellidae) (NNKN), slide D. Haelew. 955a (FH, 4 juvenile and 3 mature thalli, left elytron), reported as *Hesperomyces virescens* in Haelewaters and van Wielink (2016). ***Paratypes***: Belgium, Province Vlaams-Brabant, Meise, Domein van Bouchout, 50.927925N, 4.333069E, 28 Mar. 2019, *leg.* C. Gerstmans, on *H. sedecimguttata* (BR, CG437–CG440), slides BR5020212155379V, BR5020212156406V, BR5020212157434V, and BR5020212158462V; reported as *Hesperomyces virescens* sensu lato in De Kesel et al. (2020). *Ibid.*, 1 Apr. 2019, *leg.* C. Gerstmans, on *H. sedecimguttata* (BR, CG441–442), slides BR5020212159490V and BR5020212160236V; reported as *Hesperomyces virescens* sensu lato in De Kesel et al. (2020).

##### Description.

***Thallus*** 335–453 μm long from foot to perithecial apex; colored yellow except for a somewhat darker region right above the foot. ***Cell I*** obtriangular, 2.0–2.5× longer than broad, broadening distally, with very oblique septum I–II. ***Cell II*** longer than broad, 23–28 × 16–21 µm, subtrapezoidal in section. ***Cell III*** always smaller than cell II, 14–20 × 14–19 µm, with inflated dorsal cell wall. ***Primary appendage*** consisting of 4 superposed cells, 61–67 μm long; in the same axis as cells I and III, separated from the latter by the constricted primary septum; its basal cell somewhat longer than broad, longer than each of the remaining cells of the appendage; second to fourth cells carrying a single antherium externally, the fourth cell also carrying a second upwardly directed antherium. ***Antheridia*** flask-shaped, with slightly (dorsally and/or basally) curved efferent necks, the upper antheridium carrying at its dorsal side a pointed process, which represents the original ascospore apex. ***Cell VI*** with subparallel margins to broadening distally, 33–70 × 23–33 μm. ***Perithecium*** 194–291 × 62–86 μm (not including basal cells), symmetric or with the anterior margin convex and the posterior one almost straight or concave; broadest near the upper third, then gradually tapering towards the apex; apex complex with 2 short lower lobes, 2 upper (terminal) lobes, and 2 prominent lips surrounding the ostiole; lower lobes tapering to a rounded tip, the ventral lobe outwardly directed; terminal lobes unicellular, elongated, 29–42 μm in length, curved upwards and outwardly; ostiole with two lips, 25–29 μm in length, one lip triangular, the other slightly shorter, blunt or rounded, basally carrying the remainder of the trichogyne. ***Ascospores*** 70–85 μm long, with conspicuous slime sheath only surrounding the larger cell.

##### Material sequenced.

The Netherlands, Noord Brabant Province, Tilburg, nature reserve De Kaaistoep, 51.5333333N, 5.0166667E, 11 Aug. 2015, *leg.* H. Spijkers & P. van Wielink, on female *Halyzia sedecimguttata* (Coleoptera, Coccinellidae) (NNKN), isolate D. Haelew. 955b (7 thalli, elytra, ITS: MG757813).

##### Hosts and distribution.

On *Halyzia sedecimguttata* from Belgium and the Netherlands. Previously reported as *H. virescens* (Haelewaters and van Wielink 2016, Haelewaters et al. 2017) and *H. virescens* sensu lato (De Kesel et al. 2020). One unverified record is available from France (Justamond 2019).

##### Notes.

Supported by multi-locus phylogenetic analyses and sequence-based species delimitation methods, Haelewaters et al. (2018) showed that *H. virescens* Thaxt. is a complex of multiple species, segregated by host. The authors proposed to “restrict *H. virescens* sensu stricto to those thalli found on *Chilocorus stigma*, the host species on which the fungus was originally described” (Thaxter 1891). Here, we included two isolates from *C. stigma* (Say, 1835), and found the clade representative of *H. virescens* sensu stricto. Based on this analysis and previous work (Haelewaters et al. 2018), we can start describing the individual clades as distinct species. A monographic work with formal descriptions for the seven other species within *H. virescens* s.l. is in preparation, but in the light of this checklist we decided to describe *H. halyziae*, which was only known from a single collection in the Netherlands until we recently collected it in Belgium (Mar.–Apr. 2019).

Haelewaters and van Wielink (2016) reported an infected specimen of *Halyzia sedecimguttata* from nature reserve De Kaaistoep in the Netherlands. In 1997–2015, 476 individuals of *H. sedecimguttata* were collected on a lighted white sheet and screened for presence of Laboulbeniales, only resulting in one individual (parasite prevalence 0.2%). In Belgium, a population of infected *H. sedecimguttata* was found at the Meise Botanic Garden. Specimens were collected in spring 2019 while they were leaving their overwintering place–deep cracks in the woodwork of a small forest chapel. Screening of 46 specimens of *H. sedecimguttata* revealed nine infected ones (parasite prevalence 19.5%). This ladybird species seems to overwinter singly or in small congregations in narrow overwintering places, including in leaf litter, under foliage on stone walls, on trunks and branches (Majerus and Williams 1989). This congregation behavior is beneficial for transmission of the fungus and is also observed in *Harmonia axyridis* (Haelewaters et al. 2017).

Morphologically, *H. halyziae* is very similar to what we have thus far accepted as *H. virescens*. Within the Kingdom Fungi, there is an incredible diversity that cannot be perceived through morphology. Cryptic species are being uncovered in Agaricomycetes (e.g., Stefani et al. 2014; Sánchez-García et al. 2016), Lecanoromycetes (e.g., Singh et al. 2015), Leotiomyces (e.g., Grünig et al. 2008), Pucciniomycetes (Bennett et al. 2011), Ustilaginomycetes (e.g., Li et al. 2017), and other major clades. And while the Laboulbeniales has been the subject of a large-scale study to estimate the global species richness of the group (Weir and Hammond 1997), cryptic diversity was not part of the equation. In other words, the number of estimated species of Laboulbeniales, between 15,000 and 75,000, is likely to be corrected to include cryptic species. We note that the recognition of *H. halyziae* is only possible through molecular data and host association. Our current understanding is that, within this species complex, there is a strict parasite-host association, with one parasite found only on one host. We think that this host specificity exists at the genus level, given the *Adalia* clade (Figure 1), which includes isolates from thalli removed from two host species within the same genus.

#### Laboulbenia quarantenae

Taxon classificationFungiLaboulbenialesLaboulbeniaceae

De Kesel & Haelew. sp. nov.

05D965C7-9F36-51FB-ACB1-D37F47851B87

835490

Figure 4

##### Diagnosis.

Morphologically similar to *Laboulbenia vulgaris* Peyr., but the insertion cell is attached to the lower fifth of the posterior margin of the perithecial wall and the outer appendage is composed of 4–6(–8) branches resulting from successive dichotomies starting at the suprabasal cell, which is poorly pigmented or nearly hyaline. The LSU sequence shares 89.7–98.0% identity with other sequenced taxa of *Laboulbenia*, 97.4% with *L. flagellata* from *Agonum nigrum*, 97.5–98.0% with *L. flagellata* from *Limodromus assimilis*, 97.0–98.0% with *L. flagellata* from *Agonum emarginatum*/*A. micans*/*Loricera pilicornis*/*Oxypselaphus obscurus*, and 97.0–97.7% with *L. vulgaris* from *Bembidion tetracolum*/*Ocys harpaloides*. Unique molecular synapomorphies in the LSU at positions 503, 545.

##### Types.

***Holotype***: Belgium, Province Vlaams Brabant, Meise, Domein van Bouchout, 50.9267056N, 4.3220028E, 30 m a.s.l., 26 Apr. 2019, *leg.* A. De Kesel, rivulet-associated grassland, on Bembidion (Philochtus) biguttatum (Fabricius, 1779) (Coleoptera, Carabidae), ADK6448 (BR), slide BR5020212163329V (1 mature thallus, prothorax). Isotypes: *ibid.*, slides BR5020212162292V (2 mature thalli, right mesofemur), BR5020212161264V (6 mature thalli, right protibia), BR5020212166412V (5 immature thalli, mesothorax), BR5020212165385V (1 mature thallus, right protibia), and BR5020212164357V (1 mature thallus, right mesofemur). ***Paratype***: Belgium, Province Vlaams-Brabant, Meise, Domein van Bouchout, 50.92745N, 4.323917E, 32 m a.s.l., 30 Apr. 2020, *leg.* A. De Kesel, rivulet-associated grassland, on B. (P.) biguttatum, ADK6523 (BR), slide BR5020195033527V (2 mature thalli, mesosternum).

##### Etymology.

From *quarantena*, which was used in 14^th^–15^th^ century Venetian language for a forty-day isolation period. The new species was described during the 2020 quarantine period imposed to curb the spread of the COVID-19 virus.

##### Description.

***Thallus*** 300–465 µm long from foot to perithecial tip; colored hyaline at the lower receptacular cells (I and II) and the inner appendage, otherwise pigmented light to dark brown; especially the upper receptacular cells (III, IV and V), cell VI, and the perithecium darkening with age. ***Cell I*** elongated, usually straight, 56–107 × 22–33 µm; sometimes bent and then wider at the upper end. ***Cell II*** slender, mostly with parallel margins, longer than cell I, 73–160 × 29–40 µm, anterior margin shorter than posterior. ***Cells III and VI*** side by side, with septum II–III always much shorter than septum II–VI. Cell III with a narrow base, 29–43 µm long, widening upwards and then 22–29 µm wide at the apex. Cell VI more or less rectangular, 30–34 × 23–30 µm. ***Cell IV*** more or less rectangular, slightly broader than long, 20–32 × 25–30 µm. ***Cell V*** small, triangular, situated in the inner-upper corner of cell IV, 9–14 × 7–14 µm, as pigmented as surrounding cells. ***Insertion cell*** brownish black, flattened, barely marking a constriction on the posterior margin of the thallus, attached to the lower fifth of the posterior margin of the perithecial wall, 18–25 µm wide and 90–128 µm from the perithecial tip. ***Inner appendage*** hyaline, composed of 2–4(–6) short branches, rarely exceeding the perithecial tip, 88–150 µm long, resulting from successive dichotomies starting at the basal cell, the latter 9–14 × 6–12 µm. ***Antheridia*** short, flask-shaped, few in number, usually on the young inner appendage and arising laterally from its suprabasal cell. ***Outer appendage*** up to 250–335 µm long, extending beyond the perithecial tip, often entirely light brown, composed of 4–6(–8) branches, resulting from successive dichotomies starting at the suprabasal cell; the basal cell longer than broad, 23–32 × 15–21 µm, almost entirely hyaline. ***Perithecium*** ellipsoid, venter only very slightly asymmetrical, anterior and posterior margins almost equally convex, 109–157 × 43–64 µm, length/width ratio 1.9–2.5, widest in the middle; perithecial tip asymmetrical, with prominent and rounded posterior margin; preostiolar spots black, in older thalli merging into a pre-apical ring, always with distinctly paler zone under the posterior spot. ***Ascospores*** two-celled, hyaline, 59–65 × 4.2–5.5 µm, with slime sheath.

##### Material sequenced.

Belgium, Province Vlaams Brabant, Meise, Domein van Bouchout, 50.9267056N, 4.3220028E, 30 m a.s.l., 26 Apr. 2019, *leg.* A. De Kesel, rivulet associated grassland, on *Bembidion biguttatum* (Coleoptera, Carabidae), ADK6448 (BR), isolate E13T12 (3 mature thalli, prothorax, LSU: MT371368).

##### Hosts and distribution.

Thus far only known on *Bembidion biguttatum* from the type locality in Belgium. Reported as *Laboulbenia* sp. nov. in De Weggheleire (2019).

##### Notes.

**Figure 2.**
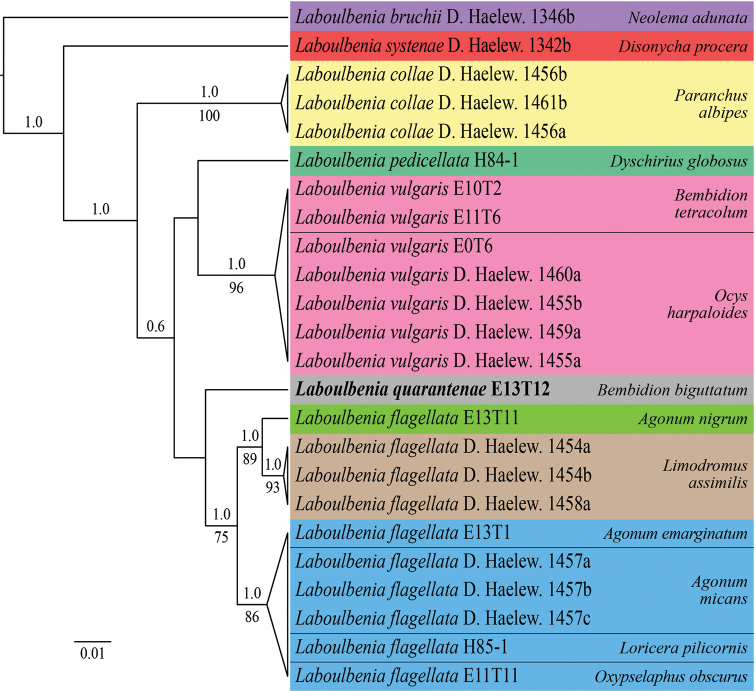
Maximum clade creditability tree of *Laboulbenia* isolates reconstructed from an LSU dataset, with *L. bruchii* as outgroup. The topology is the result of Bayesian inference performed with BEAST. For each node, ML BS (≥ 65) and Bayesian pp (≥ 0.7) are presented above/below the branch leading to that node. Isolates are color-coded by host; *L. quarantenae* sp. nov. is highlighted with gray shading.

Morphologically, *L. quarantenae* mostly resembles *L. vulgaris* Peyr., but it differs from it by the very low position of the insertion cell (regardless of the origin of the thallus), the successive dichotomous branching of the outer appendage, the poorly pigmented to nearly hyaline basal cell of the outer appendage, and the slender habitus. Although these characters may vary to some extent, eventually resulting in specimens that are morphologically close to *L. vulgaris*, our LSU phylogeny (Figure 2) shows that sequences of typical *L. vulgaris* obtained from Carabidae known to host *L. vulgaris*–*Bembidion tetracolum* Say, 1823 and *Ocys harpaloides* (Audinet-Serville, 1821) (Santamaría et al. 1991; Majewski 1994; Haelewaters et al. 2019a; De Kesel et al. 2020)–fall in a monophyletic clade separated from *L. quarantenae*. The two isolates of *L. vulgaris* from *B. tetracolum* were collected in Belgium (isolate E10T2) and Latvia (isolate E11T6), from populations that are 1,550 km apart, but they were placed together among isolates from *O. harpaloides* (all from Belgium). *Laboulbenia quarantenae*, on the other hand, was collected between <1 and 21 km distance from where hosts of *L. vulgaris* were collected.

Phylogenetically, *L. quarantenae* may be more closely related to *L. flagellata* than to *L. vulgaris*. *Laboulb quarantenae* and *L. flagellata* (sensu lato) were retrieved as sister taxa in our phylogeny, although no statistical support was retrieved for this sister relationship. Whereas species boundaries are evident based on our phylogeny, it goes without saying that both taxon sampling and sequence data need to be greatly expanded upon to resolve relationships among species of *Laboulbenia*. The new species is apparently very rare and was never found in combination with *L. vulgaris*, the more common parasite from *Bembidion biguttatum* in Belgium (De Kesel 1998; De Kesel et al. 2020).

**Figure 3.**
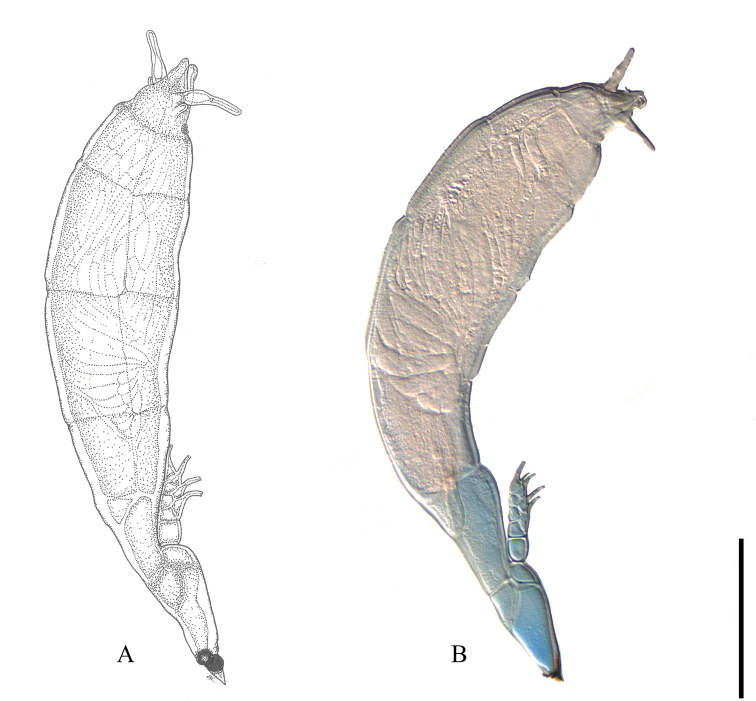
*Hesperomyces halyziae* Haelew. & De Kesel from *Halyzia sedecimguttata***A** mature thallus from slide D. Haelew. 955a, holotype **B** mature thallus from slide BR5020212156406V. Scale bar: 100 µm.

In Europe, many species of *Laboulbenia* have been reported on *Bembidion* Latreille, 1802 (Santamaría et al. 1991). Of those, *L. pedicellata* Thaxt. and *L. vulgaris* Peyr. are among the most reported ones. *Bembidion biguttatum* belongs to subfamily Trechinae. To our knowledge, this species is infected by either *L. murmanica* Huldén (S. Santamaría pers. comm.), *L. pedicellata* (Scheloske 1969; Majewski 1994), or *L. vulgaris* (Majewski 1994; De Kesel et al. 2020). Based on the position of its insertion cell as well as the morphology of both the outer appendage and the androstichum (cells II, IV, and V), *L. quarantenae* is fundamentally different from these three species. The outer appendage of *L. quarantenae* is reminiscent of the one from *L. flagellata*, which, however, is a more robust species reported from 80 genera of Carabidae belonging to Anthiinae, Brachininae, Elaphrinae, Harpalinae, Loricerinae, Nebriinae, and Patrobinae (but not Trechinae) (Santamaría et al. 1991; Santamaría 1998; Haelewaters et al. 2019a).

**Figure 4.**
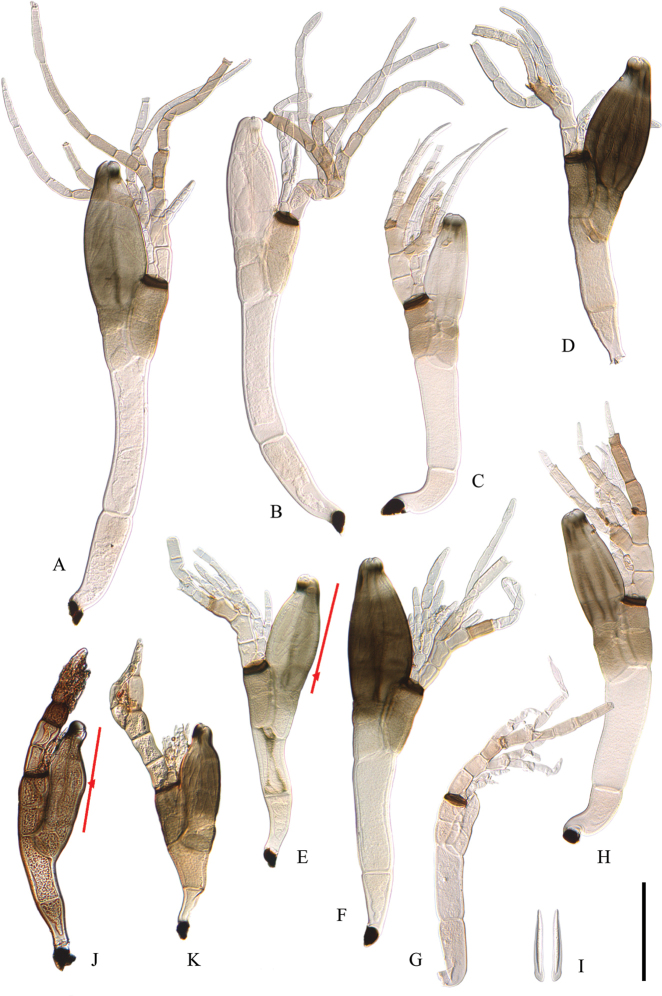
**A–I***Laboulbenia quarantena* De Kesel & Haelew. from *Bembidion biguttatum*, specimen ADK6448: **A** mature thallus from prothorax, slide BR5020212163329V, holotype **B** mature thallus from prothorax with less pigmented perithecium **C** mature thallus from the right mesofemur **D–F** mature thalli from the right protibia **G** immature thallus from the prothorax **H** mature thallus from the right mesofemur **I** ascospores **J–K** laboulbenia vulgaris Peyr: **J** mature thallus from prothorax of *Bembidion tetracolum*, specimen ADK5557 **K** mature thallus from mesothorax of *Ocys harpaloides*, specimen ADK6353. One of the diagnostic characteristics of the new species–the positioning of the insertion cell–is shown in a mature thallus of *L. quarantenae* (**E**) and one of *L. vulgaris* (**J**). Scale bar: 100 µm.

*Bembidion biguttatum*, the host for *L. quarantenae*, belongs to the subgenus Philochtus. Representatives of *Laboulbenia* reported from Bembidion subgenus Philochtus are few and include two species only: *L. pedicellata* and *L. vulgaris*. Two thalli of *Laboulbenia* “sp. similar to *L. vulgaris*” from *Bembidion bruxellense* Wesmael, 1835 [as *B. rupestre* (Linnaeus, 1767) are illustrated in Majewski (1994: Pl. 53, Figs 1, 2). Their morphology comes close to *L. quarantenae* but cell V is much larger and the insertion cell is not situated low enough along the posterior margin of the perithecial wall. Also *L. parvula* is reported on subgenus Philochtus in Santamaría et al. (1991), but this species is much smaller (180–190 µm total length) compared to *L. quarantenae*, it has a deeply pigmented basal cell of the outer appendage, the inner and outer appendage each carry 4–8 very slender branches, and its perithecial tip is rather squarish.

As we explore patterns of speciation of taxa in both Herpomycetales and Laboulbeniales using integrative taxonomy, we can start linking some of these patterns to morphological or life history traits. One candidate trait is the haustorium–a rhizoidal structure that penetrates the host’s integument to make contact with the haemocoel, increasing surface area for nutrient uptake and providing holdfast. We hypothesize that – due to the invasive nature of their haustorium – Herpomycetales and haustorial Laboulbeniales, such as species of *Hesperomyces*, maintain close interactions with their hosts, possibly involving adaptations to the hosts’ defense systems and leading to escape-and-radiate coevolution (Ehrlich and Raven 1964). These developments result in an evolutionary arms race, with specialization and leading to speciation (One Host One Parasite model, Figure 1). While all 27 species of *Herpomyces* form multiple haustoria, not all Laboulbeniales penetrate their host. Recently, Tragust et al. (2016) presented evidence for four species of Laboulbeniales to be superficially attached to their host, and also *L. flagellata* and *L. vulgaris* do not seem to perforate their hosts. There are no strict developmental barriers for non-penetrating species and their ascospores may develop on multiple arthropods given that they co-occur in a given microhabitat, resulting in parasite species with more than one host (e.g., *L. vulgaris* in Figure 2), in contrast to the host-specific species of *Hesperomyces*. Undoubtedly, other factors come into play; more studies of speciation and species limits, specificity, host shifting, and transmission patterns are needed to test said hypothesis.

### Alphabetical checklist of thallus-forming Laboulbeniomycetes in Belgium and the Netherlands

**Herpomycetales**

**1. *Herpomyces ectobiae* Thaxt., Proc. Am. Acad. Arts Sci. 38(2): 20 (1902) [1903**]

• *Blattella germanica* (Linnaeus, 1767) (Blattodea, Ectobiidae) Be

**2. *Herpomyces periplanetae* Thaxt., Proc. Am. Acad. Arts Sci. 38(2): 13 (1902) [1903**]

• *Blatta orientalis* Linnaeus, 1758 (Blattodea, Blattidae) Be

• *Periplaneta americana* (Linnaeus, 1758) (Blattodea, Blattidae) Be

**3. *Herpomyces stylopygae* Speg., Anal. Mus. Nac. Hist. Nat. B. Aires 29: 551 (1917)**

• *Blatta orientalis* Linnaeus, 1758 (Blattodea, Blattidae) Be

**Laboulbeniales**

**4. *Aphanandromyces audisioi* W. Rossi, Mycologia 74: 522 (1982)**

• *Brachypterus urticae* (Fabricius, 1792) (Coleoptera, Kateretidae) Be

**5. *Asaphomyces tubanticus* (Middelh. & Boelens) Scheloske, Parasitol. Schriftenr. 19: 92 (1969)**

• *Catops fuliginosus* Erichson, 1837 (Coleoptera, Leiodidae) Nl

• *Catops fuscus* (Panzer, 1794) Be, Nl

• *Catops longulus* Kellner, 1846 Be

• *Catops nigricans* (Spence, 1813) Be, Nl^a^

• *Catops* sp. Be

• *Choleva* sp. (Coleoptera, Leiodidae) Nl

^a^ Fungus as *Barbariella tubantica* Middelh. & Boelens ex Middelh. in Middelhoek (1949).

**6. *Bordea denotata* Haelew. & De Kesel, Nova Hedwig. 98: 114 (2014)**

• *Bibloporus bicolor* (Denny, 1825) (Coleoptera, Staphylinidae) Nl

**7. *Botryandromyces heteroceri* (Thaxt.) I.I. Tav. & T. Majewski, Mycotaxon 3: 195 (1976)**

• *Heterocerus fenestratus* (Thunberg, 1784) (Coleoptera, Heteroceridae) Be

• *Heterocerus flexuosus* Stephens, 1828 Be

• *Heterocerus hispidulus* Kiesenwetter, 1843 Be

• *Heterocerus obsoletus* Curtis, 1828 Nl

**8. *Cantharomyces denigratus* Thaxt., Mem. Am. Acad. Arts Sci. 16: 27 (1931)**

• *Dryops luridus* (Erichson, 1847) (Coleoptera, Dryopidae) Be

**9. *Cantharomyces elongatus* Haelew. & De Kesel, Mycotaxon 123: 468 (2013)**

• *Syntomium aeneum* (Müller, 1821) (Coleoptera, Staphylinidae) Nl

**10. *Cantharomyces italicus* Speg., Anal. Mus. Nac. Hist. Nat. B. Aires 27: 42 (1915)**

• *Dryops luridus* (Erichson, 1847) (Coleoptera, Dryopidae) Be

**11. *Cantharomyces orientalis* Speg., Anal. Mus. Nac. Hist. Nat. B. Aires 27: 43 (1915)**

• *Carpelimus corticinus* (Gravenhorst, 1806) (Coleoptera, Staphylinidae) Be, Nl^a^

• *Carpelimus foveolatus* (Sahlberg, 1832) Be

• *Carpelimus* sp. Be

• *Diglotta mersa* (Haliday, 1837) (Coleoptera, Staphylinidae) Be

^a^ Host as *Troglophloeus corticinus* (Gravenhorst, 1806), fungus as *Cantharomyces thaxteri* Maire in Middelhoek (1949).

**12. *Cantharomyces platystethi* Thaxt., Proc. Am. Acad. Arts Sci. 35: 415 (1900)**

• *Platystethus* sp. (Coleoptera, Staphylinidae) Be

**13. *Cantharomyces robustus* T. Majewski, Acta Mycol. 23: 99 (1990) [1987**]

• *Carpelimus bilineatus* Stephens, 1834 (Coleoptera, Staphylinidae) Be

• *Carpelimus corticinus* (Gravenhorst, 1806) Be

• *Carpelimus rivularis* (Motschulsky, 1860) Be, Nl

• *Carpelimus* sp. Be

• *Gnypeta rubrior* Tottenham, 1939 (Coleoptera, Staphylinidae) Be

**14. *Chaetarthriomyces crassiappendicatus* Scheloske**

• *Chaetarthria seminulum* (Herbst, 1797) (Coleoptera, Hydrophilidae) Nl

**15. *Chitonomyces aculeifer* Speg., Anal. Mus. Nac. Hist. Nat. B. Aires 27: 44 (1915)**

• *Graptodytes pictus* (Fabricius, 1787) (Coleoptera, Dytiscidae) Be

• *Haliplus* sp. (Coleoptera, Haliplidae) Be

**16. *Chitonomyces bidessarius* (Thaxt.) Thaxt., Mem. Am. Acad. Arts Sci. 12: 292 (1902)**

• *Hygrotus impressopunctatus* (Schaller, 1783) (Coleoptera, Dytiscidae) Nl

**17. *Chitonomyces italicus* Speg., Anal. Mus. Nac. Hist. Nat. B. Aires 27: 46 (1915)**

• *Laccophilus hyalinus* (De Geer, 1774) (Coleoptera, Dytiscidae) Be

**18. *Chitonomyces melanurus* Peyr., Sitzber. Akad. Wiss. Wien Math.-Naturw. Kl. 68: 250 (1873)**

• *Laccophilus hyalinus* (De Geer, 1774) (Coleoptera, Dytiscidae) Be

• *Laccophilus minutus* (Linnaeus, 1758) Nl^a^

^a^ New record: Utrecht Province, Soest, Soesterveen, 17 Oct. 1924, *leg.* F.C. Drescher, on *Laccophilus minutus* [as *Laccophilus obscurus* (Panzer, 1795)] (Naturalis Biodiversity Center), slide D. Haelew. 075a (BR-MYCO, 5 thalli, margin of left elytron).

**19. *Chitonomyces paradoxus* (Peyr.) Thaxt., Mem. Am. Acad. Arts Sci. 12: 287 (1902)**

• *Laccophilus hyalinus* (De Geer, 1774) (Coleoptera, Dytiscidae) Be

• *Laccophilus minutus* (Linnaeus, 1758) Nl

**20. *Compsomyces lestevae* Thaxt., Proc. Am. Acad. Arts Sci. 35: 439 (1900)**

• *Lesteva longoelytrata* (Goeze, 1777) (Coleoptera, Staphylinidae) Be

• *Lesteva pubescens* Mannerheim, 1830 Be

• Lesteva sicula subsp. heeri Fauvel, 1871 Be, Nl

• *Lesteva* sp. Be

**21. *Coreomyces arcuatus* Thaxt., Mem. Am. Acad. Arts Sci. 16: 324 (1931)**

• *Sigara striata* (Linnaeus, 1758) (Hemiptera, Corixidae) Be

**22. *Corethromyces henrotii* Balazuc [as ‘henroti’], Bull. Mens. Soc. Linn. Lyon 42: 283 (1973)**

• *Choleva cisteloides* (Frölich, 1799) (Coleoptera, Leiodidae) Be

• *Choleva fagniezi* Jeannel, 1922 Nl

• *Choleva jeanneli* Britten, 1922 Nl

• *Choleva oblonga* Latreille, 1708 Nl

**23. *Corethromyces stilici* Thaxt., Proc. Am. Acad. Arts Sci. 37: 42 (1901)**

• Rugilus (Rugilus) rufipes Germar, 1836 (Coleoptera, Staphylinidae) Be, Nl^a^

• Rugilus (Rugilus) similis (Erichson, 1839) Be

• *Rugilus* sp. Be

^a^ Host as *Stilicus rufipes* (Germar, 1836) in Middelhoek (1943a, 1945).

**24. *Cryptandromyces bibloplecti* T. Majewski, Acta Mycol. 25: 43 (1990)**

• Pselaphinae gen et sp. indet. (Coleoptera, Staphylinidae) Be

**25. *Cryptandromyces elegans* (Maire) W. Rossi & D. Castaldo, Pl. Biosystems 138: 264 (2004)**

• *Brachygluta fossulata* (Reichenbach, 1816) (Coleoptera, Staphylinidae) Nl

• *Brachygluta xanthoptera* Reichenbach, 1816 Be

**26. *Cryptandromyces euplecti* Santam., Nova Hedwig. 72: 384 (2001)**

• *Euplectus sanguineus* Denny, 1825 (Coleoptera, Staphylinidae) Be

**27. *Dimorphomyces myrmedoniae* Thaxt., Proc. Am. Acad. Arts Sci. 36: 409 (1900) [1901**]

• *Gnypeta rubrior* Tottenham, 1939 (Coleoptera, Staphylinidae) Be

**28. *Diphymyces kaaistoepi* Haelew. & De Kesel, Sterbeeckia 35: 63 (2019)**

• *Choleva cisteloides* (Frölich, 1799) (Coleoptera, Leiodidae) Be

• *Choleva fagniezi* Jeannel, 1922 Nl

**29. *Distolomyces forficulae* (T. Majewski) I.I. Tav., Mycol. Mem. 9: 207 (1985)**

• *Forficula auricularia* Linnaeus, 1758 (Dermaptera, Forficulidae) Be, Nl

**30. *Ecteinomyces trichopterophilus* Thaxt., Proc. Am. Acad. Arts Sci. 38: 26 (1902) [1903**]

• *Acrotrichis fascicularis* (Herbst, 1793) (Coleoptera, Ptiliidae) Be

• *Acrotrichis grandicollis* (Mannerheim, 1844) Nl

• *Acrotrichis intermedia* (Gillmeister, 1845) Be

• *Acrotrichis* sp. Be

**31. *Eucantharomyces stammeri* Scheloske, Parasitol. Schriftenr. 19: 108 (1969)**

• *Calathus melanocephalus* (Linnaeus, 1758) (Coleoptera, Carabidae) Be

**32. *Euphoriomyces agathidii* (Maire) I.I. Tav., Mycol. Mem. 9: 218 (1985)**

• *Agathidium laevigatum* Erichson, 1845 (Coleoptera, Leiodidae) Nl^a^

^a^ New record: Noord Brabant Province, Tilburg, nature reserve De Kaaistoep, 51.540672N 5.013867E, 3–17 Jun. 2000, *leg.* Working Group Insects of the Royal Dutch Natural History Association (KNNV), pitfall trap, ±2.5 m S of *Quercus robur* #2, on *Agathidium laevigatum* (NNKN), slides D. Haelew. 1064a (FH, 1 submature and 2 mature thalli, tip of left elytron) and D. Haelew. 1064b (NMBT, 1 juvenile and 2 mature thalli, tip of right elytron).

**33. *Euzodiomyces lathrobii* Thaxt., Proc. Am. Acad. Arts Sci. 35: 449 (1900)**

• *Lathrobium brunnipes* (Fabricius, 1793) (Coleoptera, Staphylinidae) Be

• *Lathrobium elongatum* (Linnaeus, 1767) Be, Nl

• *Lathrobium geminum* Kraatz, 1857 Be, Nl

• *Lathrobium laevipenne* Heer, 1839 Nl

• *Lathrobium* sp. Be

• *Lobrathium multipunctum* (Gravenhorst, 1802) (Coleoptera, Staphylinidae) Be

• *Patrobus atrorufus* (Stroem, 1768) (Coleoptera, Carabidae) Be

• *Pterostichus strenuus* (Panzer, 1796) (Coleoptera, Carabidae) Be

**34. *Fanniomyces burdigalensis* Balazuc, Revue Mycol. 43: 402 (1979)**

• *Copromyza stercoraria* (Meigen, 1830) (Diptera, Sphaeroceridae) Be^a^

• *Crumomyia pedestris* (Meigen, 1830) (Diptera, Sphaeroceridae) Be^a^

^a^ Fungus as *Stigmatomyces burdigalensis* (Balazuc) A. Weir & W. Rossi in De Kesel et al. (2020).

**35. *Fanniomyces ceratophorus* (Whisler) T. Majewski, Acta Mycol. 8: 230 (1972)**

• *Fannia canicularis* (Linnaeus, 1761) (Diptera, Fanniidae) Nl^a^

^a^ Fungus described as *Stigmatomyces ceratophorus* Whisler, and later recombined in *Fanniomyces* T. Majewski by Majewski (1972), based on the branching pattern of the primary appendage. Weir and Rossi (1995), in turn, found no valid rationale to maintain *Fanniomyces* as a separate genus and considered it a junior synonym of *Stigmatomyces*, stating that “the structure of the antheridial appendage is particularly variable”. However, based on an SSU–LSU ribosomal DNA dataset, Haelewaters et al. (in press) found that 1) *Stigmatomyces* as currently circumscribed is paraphyletic and 2) *Fanniomyces* is supported as a stand-alone genus with two species, *F. burdigalensis* and *F. ceratophorus*.

**36. *Haplomyces texanus* Thaxt., Proc. Am. Acad. Arts Sci. 28: 160 (1893)**

• *Bledius gallicus* (Gravenhorst, 1806) (Coleoptera, Staphylinidae) Nl^a^

^a^ Host as *Bledius fracticornis* (Paykull, 1790) in Middelhoek (1943a).

**37. *Helodiomyces elegans* F. Picard, Bull. Soc. Mycol. Fr. 29: 557 (1913)**

• *Dryops anglicanus* Edwards, 1909 (Coleoptera, Dryopidae) Nl

• *Dryops auriculatus* (Geoffroy, 1785) Nl

• *Dryops luridus* (Erichson, 1847) Be, Nl

**38. *Hesperomyces coccinelloides* Thaxt., Mem. Am. Acad. Arts Sci. 16: 110 (1931)**

• *Stethorus punctillum* (Weise, 1891) (Coleoptera, Coccinellidae) Be

**39. *Hesperomyces halyziae* Haelew. & De Kesel, sp. nov.**

• *Halyzia sedecimguttata* (Linnaeus, 1758) (Coleoptera, Coccinellidae) Be^a^, Nl^b^

^a^ Fungus as *Hesperomyces virescens* Thaxt. sensu lato in De Kesel et al. (2020).

^b^ Fungus as *Hesperomyces virescens* Thaxt. in Haelewaters and van Wielink (2016) and Haelewaters et al. (2017).

**40. *Hesperomyces virescens* Thaxt., Proc. Am. Acad. Arts Sci. 25: 264 (1891), sensu lato**

• *Harmonia axyridis* (Pallas, 1773) (Coleoptera, Coccinellidae) Be, Nl

• *Tytthaspis sedecimpunctata* (Linnaeus, 1761) (Coleoptera, Coccinellidae) Be

**41. *Hydraeomyces halipli* (Thaxt.) Thaxt., Mem. Am. Acad. Arts Sci. 12: 294 (1902)**

• *Haliplus flavicollis* Sturm, 1834 (Coleoptera, Haliplidae) Nl

• *Haliplus immaculatus* Gerhardt, 1877 Be

• *Haliplus lineatocollis* (Marsham, 1802) Be

• *Haliplus lineolatus* Mannerheim, 1844 Be

• *Haliplus ruficollis* (De Geer, 1774) Be, Nl

• *Haliplus* sp. Be

**42. Hydrophilomyces cf. gracilis T. Majewski, Acta Mycol. 10: 272 (1974)**

• *Cercyon marinus* Thomson, 1853 (Coleoptera, Hydrophilidae) Be

• *Cercyon* sp. Be

**43. Hydrophilomyces cf. hamatus T. Majewski, Acta Mycol. 10: 274 (1974)**

• *Cercyon marinus* Thomson, 1853 (Coleoptera, Hydrophilidae) Be

**44. *Idiomyces peyritschii* Thaxt., Proc. Am. Acad. Arts Sci. 28: 162 (1893)**

• *Deleaster dichrous* Gravenhorst, 1802 (Coleoptera, Staphylinidae) Be, Nl

**45. *Kainomyces rehmanii* T. Majewski, Polish Bot. Stud. 1: 121 (1990)**

• *Acrotrichis dispar* (Matthews, 1865) (Coleoptera, Ptiliidae) Nl

• *Acrotrichis* sp. Be

**46. *Laboulbenia acupalpi* Speg., Anal. Mus. Nac. Hist. Nat. B. Aires 26: 458 (1915)**

• *Acupalpus parvulus* (Sturm, 1825) (Coleoptera, Carabidae) Nl

**47. *Laboulbenia anoplogenii* Thaxt., Proc. Am. Acad. Arts Sci. 35: 156 (1899) [1899–1900**]

• *Stenolophus mixtus* (Herbst, 1784) (Coleoptera, Carabidae) Be, Nl

• *Stenolophus teutonus* (Schrank, 1781) Be

**48. *Laboulbenia argutoris* Cépède & F. Picard, Bull. Biol. Fr. Belg. 42: 260 (1909)**

• *Pterostichus diligens* (Sturm, 1824) (Coleoptera, Carabidae) Be

• *Pterostichus strenuus* (Panzer, 1796) Be, Nl

• *Pterostichus vernalis* (Panzer, 1796) Nl

**49. *Laboulbenia atlantica* Thaxt., Mem. Am. Acad. Arts Sci. 12: 336 (1902)**

• *Lobrathium multipunctum* (Gravenhorst, 1802) (Coleoptera, Staphylinidae) Be

**50. *Laboulbenia aubryi* Balazuc, Revue Mycol. 43: 393 (1979)**

• *Amara aenea* (De Geer, 1774) (Coleoptera, Carabidae) Be^a^

^a^ New record: Belgium, Province Vlaams Brabant, Meise, Domein van Bouchout, 50.9274389N 4.323925E, ca. 25 m a.s.l., 6 Apr. 2020, leg. A. De Kesel, wet meadow, on *Amara aenea*, ADK6520 (BR), slides ADK6520a (BR-MYCO, 1 mature thallus, elytra) and ADK6520b (BR-MYCO, 2 immature and 4 mature thalli, elytra).

**51. *Laboulbenia barbara* Middelh. & Boelens, Ned. Kruidk. Arch. 53: 99 (1943a)**

• *Philonthus punctus* (Gravenhorst, 1802) (Coleoptera, Staphylinidae) Nl

**52. *Laboulbenia benjaminii* Balazuc ex Santam., Fl. Mycol. Iber. 4: 45 (1998)**

• *Badister bullatus* (Schrank, 1798) (Coleoptera, Carabidae) Be, Nl^a^

• *Badister lacertosus* Sturm, 1815 Be

• *Badister sodalis* (Duftschmid, 1812) Be, Nl^b^

• *Badister unipustulatus* Bonelli, 1813 Be

^a^ Host as *Badister bipustulatus* (Fabricius, 1792), fungus as *Laboulbenia polyphaga* Thaxt. in Middelhoek (1949) and Meijer (1975).

^b^ Fungus as *Laboulbenia polyphaga* Thaxt. in Meijer (1975).

**53. *Laboulbenia calathi* T. Majewski, Polish Bot. Stud. 7: 89 (1994)**

• *Calathus erratus* (Sahlberg, 1827) (Coleoptera, Carabidae) Be

• *Calathus fuscipes* (Goeze, 1777) Nl

• *Calathus melanocephalus* (Linnaeus, 1758) Be, Nl

**54. *Laboulbenia clivinalis* Thaxt., Proc. Am. Acad. Arts Sci. 35: 155 (1899) [1899–1900**]

• *Clivina collaris* (Herbst, 1784) (Coleoptera, Carabidae) Be

• *Clivina fossor* (Linnaeus, 1758) Be, Nl

**55. *Laboulbenia collae* T. Majewski, Polish Bot. Stud. 7: 104 (1994)**

• *Agonum micans* (Nicolai, 1822) (Coleoptera, Carabidae) Be

• *Paranchus albipes* (Fabricius, 1796) (Coleoptera, Carabidae) Be, Nl

**56. *Laboulbenia coneglianensis* Speg., Redia 10: 47 (1914)**

• *Harpalus affinis* (Schrank, 1781) (Coleoptera, Carabidae) Be, Nl^a^

• *Harpalus atratus* Latreille, 1804 Be

• *Harpalus attenuatus* Stephens, 1828 Be

• *Harpalus griseus* (Panzer, 1796) Be, Nl^b^

• *Harpalus rufipes* (De Geer, 1774) Be

• *Harpalus tardus* (Panzer, 1796) Be, Nl

• *Harpalus* sp. Be

• *Ophonus rufibarbis* (Fabricius, 1792) (Coleoptera, Carabidae) Be

• *Parophonus maculicornis* (Duftschmid, 1812) (Coleoptera, Carabidae) Nl^c^

^a^ Host as *Harpalus aeneus* (Fabricius, 1775), fungus as *Laboulbenia elongata* Thaxt. in Middelhoek (1949).

^b^ Host as *Pseudophonus griseus* (Panzer, 1796), fungus as *Laboulbenia elongata* Thaxt. in Middelhoek (1949).

^c^ Fungus as *Laboulbenia melanaria* Thaxt. in Haelewaters et al. (2012a).

**57. *Laboulbenia cristata* Thaxt., Proc. Am. Acad. Arts Sci. 29: 174 (1893)**

• *Paederus fuscipes* Curtis, 1826 (Coleoptera, Staphylinidae) Nl

• *Paederus littoralis* Gravenhorst, 1802 Be

• *Paederus riparius* (Linnaeus, 1758) Be, Nl

• *Paederus* sp. Be

**58. *Laboulbenia dubia* Thaxt., Proc. Am. Acad. Arts Sci. 38: 35 (1902) [1903**]

• *Philonthus cognatus* Stephens, 1832 (Coleoptera, Staphylinidae) Be

**59. *Laboulbenia egens* Speg., Anal. Soc. Cient. Argent. 85: 323 (1918)**

• *Elaphropus parvulus* (Dejean, 1831) (Coleoptera, Carabidae) Be

• *Paratachys micros* (Fischer von Waldheim, 1828) (Coleoptera, Carabidae) Be

**60. *Laboulbenia elaphri* Speg., Anal. Mus. Nac. B. Aires 26: 64 (1915)**

• *Elaphrus cupreus* Duftschmid, 1812 (Coleoptera, Carabidae) Be

• *Elaphrus riparius* (Linnaeus, 1758) Be

**61. *Laboulbenia eubradycelli* Huldén, Karstenia 25: 4 (1985)**

• *Bradycellus harpalinus* (Audinet-Serville, 1821) (Coleoptera, Carabidae) Be, Nl

• *Bradycellus ruficollis* (Stephens, 1828) Be

• *Bradycellus verbasci* (Duftschmid, 1812) Be, Nl

• *Trichocellus placidus* (Gyllenhal, 1827) (Coleoptera, Carabidae) Be

**62. *Laboulbenia fasciculata* Peyr., Sitzber. Akad. Wiss. Wien Math.-naturw. Kl. 68: 248 (1873)**

• *Nebria brevicollis* (Fabricius, 1792) (Coleoptera, Carabidae) Be

• *Omophron limbatum* (Fabricius, 1777) (Coleoptera, Carabidae) Be, Nl^a^

• *Patrobus atrorufus* (Stroem, 1768) (Coleoptera, Carabidae) Be

• *Pterostichus nigrita* (Paykull, 1790) (Coleoptera, Carabidae) Be

^a^ New record: No locality, no date, on *Omophron limbatum* (Naturalis Biodiversity Center), slide D. Haelew. 074a (BR-MYCO, 3 thalli, left metatibia).

**63. *Laboulbenia fennica* Huldén, Karstenia 23: 54 (1983)**

• *Gyrinus marinus* Gyllenhal, 1808 (Coleoptera, Gyrinidae) Nl

• *Gyrinus substriatus* Stephens, 1829 Be, Nl

**64. *Laboulbenia filifera* Thaxt., Proc. Am. Acad. Arts Sci. 28: 165 (1893)**

• *Harpalus affinis* (Schrank, 1781) (Coleoptera, Carabidae) Nl^a^

^a^ Host as *Harpalus aeneus* (Fabricius, 1775) in Middelhoek (1949). The microscope slide from the collection of W.J. Kossen was reported to be in very poor condition; as a result, no illustrations could be made (Middelhoek 1949). For the time being, we retain the identification of the fungus. *Laboulbenia filifera* was described on a species of *Anisodactylus* Dejean, 1829 (Coleoptera, Carabidae) in the USA, and it is possible that European records of *L. filifera* belong in *L. flagellata* (Majewski 1994, Haelewaters et al. 2019a). The species is not included in the identification key.

**65. *Laboulbenia flagellata* Peyr., Sitzber. Akad. Wiss. Wien Math.-naturw. Kl. 68: 247 (1873), sensu lato**

• *Agonum emarginatum* (Gyllenhal, 1827) (Coleoptera, Carabidae) Be

• *Acupalpus flavicollis* (Sturm, 1825) Nl^a^

• *Agonum fuliginosum* (Panzer, 1809) Be, Nl^b^

• *Agonum marginatum* (Linnaeus, 1758) Be, Nl

• *Agonum micans* (Nicolai, 1822) Be

• *Agonum moestum* (Duftschmid, 1812) Be, Nl^a^

• *Agonum muelleri* (Herbst, 1784) Be, Nl

• *Agonum nigrum* Dejean, 1828 Be

• *Agonum thoreyi* Dejean, 1828 Be, Nl

• *Agonum viduum* (Panzer, 1796) Nl

• *Agonum viridicupreum* Goeze, 1777 Be

• *Anchomenus dorsalis* (Pontoppidan, 1763) (Coleoptera, Carabidae) Nl^c^

• *Anisodactylus binotatus* (Fabricius, 1787) (Coleoptera, Carabidae) Be

• *Laemostenus terricola* (Herbst, 1784) (Coleoptera, Carabidae) Be

• *Limodromus assimilis* (Paykull, 1790) (Coleoptera, Carabidae) Be, Nl^d^

• *Loricera pilicornis* (Fabricius, 1775) (Coleoptera, Carabidae) Be

• *Nebria brevicollis* (Fabricius, 1792) (Coleoptera, Carabidae) Be

• *Oxypselaphus obscurus* (Herbst, 1784) (Coleoptera, Carabidae) Be

• *Paranchus albipes* (Fabricius, 1796) (Coleoptera, Carabidae) Be, Nl^e^

• *Parophonus maculicornis* (Duftschmid, 1812) Be

• *Pterostichus vernalis* (Panzer, 1796) Be

• *Trichotichnus laevicollis* (Duftschmid, 1812) (Coleoptera, Carabidae) Be

^a^ Fungus as *Laboulbenia elongata* Thaxt. in Middelhoek (1949).

^b^ Host as *Europhilus fuliginosus* (Panzer, 1809), fungus as *Laboulbenia elongata* Thaxt. in Middelhoek (1949).

^c^ Host as *Platynus dorsalis* (Pontoppidan, 1763) in Zaneveld (1938), as *Agonum dorsale* (Pontoppidan, 1763) in Meijer (1975).

^d^ Host as *Platynus assimilis* (Paykull, 1790) in Zaneveld (1938).

^e^ Host as *Platynus ruficornis* (Goeze, 1777) in Zaneveld (1938).

**66. *Laboulbenia giardii* Cépède & F. Picard, Bull. Sci. Fr. Belg. 42: 258 (1908)**

• *Dicheirotrichus gustavii* Crotch, 1871 (Coleoptera, Carabidae) Be, Nl^a^

• *Dicheirotrichus obsoletus* (Dejean, 1829) Be

^a^ Host as *Dicheirotrichus pubescens* (Paykull, 1790) in Meijer (1975).

**67. *Laboulbenia gyrinicola* Speg., Redia 10: 34 (1914)**

• *Gyrinus marinus* Gyllenhal, 1808 (Coleoptera, Gyrinidae) Be, Nl

• *Gyrinus natator* (Linnaeus, 1758) Be

• *Gyrinus substriatus* Stephens, 1829 Nl

**68. *Laboulbenia hyalopoda* De Kesel, Sterbeeckia 18: 17 (1998)**

• *Paradromius linearis* (Olivier, 1795) (Coleoptera, Carabidae) Be

**69. *Laboulbenia inflata* Thaxt., Proc. Am. Acad. Arts Sci. 27: 41 (1892)**

• *Acupalpus dubius* Schilsky, 1888 (Coleoptera, Carabidae) Be, Nl

• *Acupalpus exiguus* Dejean, 1829 Be

• *Acupalpus parvulus* (Sturm, 1825) Nl

• *Stenolophus mixtus* (Herbst, 1784) (Coleoptera, Carabidae) Be

**70. *Laboulbenia kajanensis* Huldén, Karstenia 23: 56 (1983)**

• *Pterostichus diligens* (Sturm, 1824) (Coleoptera, Carabidae) Be

• *Pterostichus strenuus* (Panzer, 1796) Be

**71. *Laboulbenia lecoareri* (Balazuc) Huldén, Karstenia 25: 6 (1985)**

• *Trechoblemus micros* (Herbst, 1784) (Coleoptera, Carabidae) Be

**72. *Laboulbenia leisti* J. Siemaszko & Siemaszko, Polsk. Pism. Entomol. 6: 203 (1928) [1927**]

• *Agonum muelleri* (Herbst, 1784) (Coleoptera, Carabidae) Be

• *Leistus ferrugineus* (Linnaeus, 1758) (Coleoptera, Carabidae) Be, Nl

**73. *Laboulbenia lichtensteinii* F. Picard, Bull. Sci. Fr. Belg. 50: 449 (1917) [1916–1917**]

• *Cillenus lateralis* Samouelle, 1819 (Coleoptera, Carabidae) Nl

**74. *Laboulbenia littoralis* De Kesel & Haelew., Mycologia 106: 408 (2014)**

• *Cafius xantholoma* (Gravenhorst, 1806) (Coleoptera, Staphylinidae) Be, Nl

**75. *Laboulbenia luxurians* Peyr., Sitzber. Akad. Wiss. Wien Math.-naturw. Kl. 68: 248 (1873)**

• *Bembidion dentellum* (Thunberg, 1787) (Coleoptera, Carabidae) Nl

**76. *Laboulbenia metableti* Scheloske, Parasitol. Schriftenr. 19: 124 (1969)**

• *Syntomus foveatus* (Geoffroy, 1785) (Coleoptera, Carabidae) Be, Nl^a^

• *Syntomus truncatellus* (Linnaeus, 1760) Be, Nl^a^

^a^ New records: Noord-Holland Province, Zuid-Kennemerland National Park, 31 Oct. 2016, *leg.* M. Boeken, pitfall trap, on *Syntomus truncatellus*, slide D. Haelew. 1236b (GENT, 2 juvenile thalli, pronotum). *Ibid.*, 5 Jun. 2017, *leg.* M. Boeken, pitfall trap, on *Syntomus truncatellus*, slide D. Haelew. 1378a (GENT, 2 mature thalli, posterior margin of right elytron). *Ibid.*, 5 Jun. 2017, *leg.* M. Boeken, pitfall trap, on *Syntomus foveatus*, slide D. Haelew. 1387a (GENT, 1 mature thallus, left elytron). *Ibid.*, 5 Jun. 2017, *leg.* M. Boeken, pitfall trap, on *Syntomus foveatus*, slides D. Haelew. 1391a (FH, 5 mature thalli, right elytron), D. Haelew. 1391b (FH, 1 mature thallus, left metatrochanter), and D. Haelew. 1391c (FH, 1 submature and 2 mature thalli, mesocoxae). *Ibid.*, 17 Jul. 2017, *leg.* M. Boeken, pitfall trap, on *Syntomus truncatellus*, slide D. Haelew. 1379a (GENT, 2 juvenile thalli, left elytron).

**77. *Laboulbenia murmanica* Huldén, Karstenia 23: 57 (1983)**

• *Bembidion assimile* Gyllenhal, 1810 (Coleoptera, Carabidae) Be

**78. *Laboulbenia notiophili* Cépède & F. Picard, Bull. Biol. Fr. Belg. 42: 259 (1909)**

• *Demetrias atricapillus* (Linnaeus, 1758) (Coleoptera, Carabidae) Be

• *Demetrias imperialis* (Germar, 1824) Be

• *Demetrias monostigma* Leach, 1819 Be

• *Notiophilus biguttatus* (Fabricius, 1779) (Coleoptera, Carabidae) Be, Nl

• *Notiophilus rufipes* Curtis, 1829 Be

• *Notiophilus substriatus* Waterhouse, 1833 Nl

• *Notiophilus* sp. Be

• *Paradromius linearis* (Olivier, 1795) (Coleoptera, Carabidae) Be, Nl^a^

• *Philorhizus melanocephalus* (Dejean, 1825) (Coleoptera, Carabidae) Nl

^a^ Fungus as *Laboulbenia casnoniae* Thaxt. in Haelewaters et al. (2012a).

**79. *Laboulbenia ophoni* Thaxt., Proc. Am. Acad. Arts Sci. 35: 190 (1899) [1899–1900**]

• *Harpalus rubripes* (Duftschmid, 1812) (Coleoptera, Carabidae) Be

• *Ophonus rufibarbis* (Fabricius, 1792) (Coleoptera, Carabidae) Be

**80. *Laboulbenia pedicellata* Thaxt., Proc. Am. Acad. Arts Sci. 29: 109 (1893)**

• *Bembidion aeneum* Germar, 1824 (Coleoptera, Carabidae) Be, Nl

• *Bembidion articulatum* (Panzer, 1796) Nl

• *Bembidion biguttatum* (Fabricius, 1779) Nl

• *Bembidion gilvipes* Sturm, 1825 Be

• *Bembidion guttula* (Fabricius, 1792) Be, Nl

• *Bembidion iricolor* Bedel, 1879 Be, Nl

• *Bembidion lunulatum* (Geoffroy, 1785) Be, Nl

• *Bembidion minimum* (Fabricius, 1792) Be, Nl

• *Bembidion normannum* Dejean, 1831 Be, Nl

• *Bembidion obtusum* Audinet-Serville, 1821 Be

• *Bembidion quadrimaculatum* (Linnaeus, 1760) Be, Nl

• *Bembidion ustulatum* (Linnaeus, 1758) Nl

• *Bembidion varium* (Olivier, 1795) Be, Nl

• *Dyschirius globosus* (Herbst, 1784) (Coleoptera, Carabidae) Nl

• *Dyschirius salinus* Schaum, 1843 Nl

• *Dyschirius thoracicus* (P. Rossi, 1790) Nl^a^

• *Dyschirius tristis* Stephens, 1827 Be

• *Dyschirius* sp. Nl

• *Pogonus chalceus* (Marsham, 1802) (Coleoptera, Carabidae) Be, Nl

^a^ Host as *Dyschirius arenosus* Stephens, 1827 in Middelhoek (1943a).

**81. *Laboulbenia philonthi* Thaxt., Proc. Am. Acad. Arts Sci. 28: 174 (1893)**

• *Philonthus micans* (Gravenhorst, 1802) (Coleoptera, Staphylinidae) Nl

• *Philonthus rubripennis* Stephens, 1832 (Coleoptera, Staphylinidae) Be

• *Philonthus* sp. Be

**82. *Laboulbenia pseudomasei* Thaxt., Proc. Am. Acad. Arts Sci. 35: 196 (1899)**

• *Loricera pilicornis* (Fabricius, 1775) (Coleoptera, Carabidae) Be

• *Nebria brevicollis* (Fabricius, 1792) (Coleoptera, Carabidae) Be

• *Pterostichus anthracinus* (Panzer, 1795) (Coleoptera, Carabidae) Be

• *Pterostichus melanarius* (Illiger, 1798) Nl^a^

• *Pterostichus minor* (Gyllenhal, 1827) Be

• *Pterostichus nigrita* (Paykull, 1790) Be

• *Pterostichus strenuus* (Panzer, 1796) Be

• *Stomis pumicatus* (Panzer, 1796) (Coleoptera, Carabidae) Be

^a^ New record: Drenthe Province, Oude Willem, 52.897438N 6.323432E, 2 Jun. 2014, *leg.* A.J. Dees, on *Pterostichus melanarius* (NNKN), slides D. Haelew. 1013a (FH, 1 juvenile thallus, right elytron) and D. Haelew. 1013b (FH, 1 submature thallus, prosternum).

**83. *Laboulbenia quarantenae* De Kesel & Haelew, sp. nov.**

• Bembidion (Philochtus) biguttatum (Fabricius, 1779) (Coleoptera, Carabidae) Be

**84. *Laboulbenia rougetii* Mont. & C.P. Robin, in Robin, *Histoire Naturelle des végétaux parasites qui croissent sur l’homme et sur les animaux vivants* (Paris): 622 (1853)**

• *Brachinus crepitans* (Linnaeus, 1758) (Coleoptera, Carabidae) Be

**85. *Laboulbenia slackensis* Cépède & F. Picard, Compt. Rend. Assoc. Franç. Avancem. Sci. 35: 775 (1907)**

• *Pogonus chalceus* (Marsham, 1802) (Coleoptera, Carabidae) Be, Nl

**86. *Laboulbenia stilicicola* Speg., Redia 10: 41 (1914)**

• *Rugilus orbiculatus* (Paykull, 1789) (Coleoptera, Staphylinidae) Be, Nl^a^

• *Rugilus rufipes* Germar, 1836 (Coleoptera, Staphylinidae) Be, Nl^b^

^a^ Host as *Stilicus orbiculatus* (Paykull, 1789), fungus as *Laboulbenia subterranea* Thaxt. in Middelhoek (1943a, 1947a).

^b^ Host as *Stilicus rufipes* Germar, 1836, fungus as *Laboulbenia subterranea* Thaxt. in Middelhoek (1943a, 1945).

**87. *Laboulbenia thaxteri* Cépède & F. Picard, Bull. Biol. Fr. Belg. 42: 260 (1909)**

• *Asaphidion flavipes* (Linnaeus, 1760) (Coleoptera, Carabidae) Be

**88. *Laboulbenia vulgaris* Peyr., Sitzber. Akad. Wiss. Wien Math.-naturw. Kl. 68: 248 (1873)**

• *Asaphidion flavipes* (Linnaeus, 1760) (Coleoptera, Carabidae) Nl

• *Bembidion assimile* Gyllenhal, 1810 (Coleoptera, Carabidae) Nl

• *Bembidion biguttatum* (Fabricius, 1779) Be, Nl

• *Bembidion bruxellense* Wesmael, 1835 Nl^a^

• *Bembidion dentellum* (Thunberg, 1787) Be, Nl

• *Bembidion elongatum* Dejean, 1831 Be

• *Bembidion femoratum* Sturm, 1825 Be, Nl

• *Bembidion iricolor* Bedel, 1879 Nl

• *Bembidion mannerheimi* Sahlberg, 1827 Be

• *Bembidion minimum* (Fabricius, 1792) Nl

• *Bembidion normannum* Dejean, 1831 Nl

• *Bembidion pallidipenne* (Illiger, 1802) Nl

• *Bembidion properans* (Stephens, 1828) Be, Nl

• *Bembidion stephensii* Crotch, 1866 Be

• *Bembidion testaceum* (Duftschmid, 1812) Nl

• *Bembidion tetracolum* Say, 1823 Be, Nl

• *Bembidion tibiale* (Duftschmid, 1812) Be

• *Bembidion ustulatum* (Linnaeus, 1758) Nl

• *Bembidion* sp. Be

• *Dyschirius globosus* (Herbst, 1784) (Coleoptera, Carabidae) Nl

• *Dyschirius salinus* Schaum, 1843 Nl

• *Ocys harpaloides* (Audinet-Serville, 1821) (Coleoptera, Carabidae) Be

• *Trechus quadristriatus* (Schrank, 1781) (Coleoptera, Carabidae) Be

• *Trechus rubens* (Fabricius, 1792) Be

^a^ Host as *Bembidion rupestre* (Linnaeus, 1767) in Meijer (1975).

**89. *Mimeomyces zeelandicus* Middelh. & Boelens, Ned. Kruidk. Arch. 53: 102 (1943)**

• *Heterothops binotatus* (Gravenhorst, 1802) (Coleoptera, Staphylinidae) Nl

**90. *Misgomyces dyschirii* Thaxt., Proc. Am. Acad. Arts Sci. 35: 443 (1900)**

• *Dyschirius aeneus* (Dejean, 1825) (Coleoptera, Carabidae) Be, Nl

• *Dyschirius globosus* (Herbst, 1784) Be, Nl

• *Dyschirius intermedius* Putzeys, 1846 Be

• *Dyschirius politus* (Dejean, 1825) Nl

• *Dyschirius salinus* Schaum, 1843 Nl

• *Dyschirius tristis* Stephens, 1827 Be, Nl^a^

^a^ Host as *Dyschirius luedersi* Wagner, 1915 in Middelhoek (1943a).

**91. *Monoicomyces bolitocharae* T. Majewski, Polish Bot. Stud. 7: 193 (1994)**

• *Bolitochara obliqua* Erichson, 1837 (Coleoptera, Staphylinidae) Be

**92. *Monoicomyces britannicus* Thaxt., Proc. Am. Acad. Arts Sci. 35: 412 (1900)**

• *Acrotona fungi* (Gravenhorst, 1806) (Coleoptera, Staphylinidae) Be^a^

• *Acrotona orbata* (Erichson, 1837) Be^b^

• *Acrotona pseudotenera* (Cameron, 1933) Nl

• *Atheta* sp. (Coleoptera, Staphylinidae) Be

^a^ Host as Atheta (Mocyta) fungi (Gravenhorst, 1806) in De Kesel et al. (2020).

^b^ Host as Atheta (Mocyta) orbata (Erichson, 1837) in De Kesel et al. (2020).

**93. *Monoicomyces californicus* (Thaxt.) Thaxt., Mem. Am. Acad. Arts Sci. 16: 38 (1931)**

• *Anotylus sculpturatus* (Gravenhorst, 1806) (Coleoptera, Staphylinidae) Be, Nl^a^

^a^ Host as *Oxytelus sculpturatus* Gravenhorst, 1806 in Middelhoek (1943a).

**94. *Monoicomyces fragilis* Scheloske, Parasitol. Schriftenr. 19: 138 (1969)**

• *Ocalea picata* (Stephens, 1832) (Coleoptera, Staphylinidae) Be

**95. *Monoicomyces homalotae* Thaxt., Proc. Am. Acad. Arts Sci. 35: 412 (1900)**

• *Atheta aeneicollis* (Sharp, 1869) (Coleoptera, Staphylinidae) Nl

• *Atheta amicula* (Stephens, 1832) Nl

• *Atheta crassicornis* (Fabricius, 1792) Nl

• *Atheta gagatina* (Baudi, 1848) Nl

• *Atheta longicornis* (Gravenhorst, 1802) Be

• *Atheta triangulum* (Kraatz, 1856) Be, Nl

• *Atheta vestita* (Gravenhorst, 1806) Be

• *Atheta xanthopus* (Thomson, 1856) Nl

• *Atheta* sp. Be

**96. *Monoicomyces invisibilis* Thaxt., Proc. Am. Acad. Arts Sci. 36: 414 (1900) [1901**]

• *Anotylus sculpturatus* (Gravenhorst, 1806) (Coleoptera, Staphylinidae) Be

• *Anotylus* sp. Be

• *Oxytelus laqueatus* (Marsham, 1802) (Coleoptera, Staphylinidae) Be

• *Oxytelus* sp. Be

• *Platystethus arenarius* (Geoffroy, 1785) (Coleoptera, Staphylinidae) Be

**97. *Monoicomyces matthiatis* T. Majewski, Acta Mycol. 25: 49 (1990) [1989**]

• Platystethus cf. arenarius (Geoffroy, 1785) (Coleoptera, Staphylinidae) Be

**98. *Monoicomyces myllaenae* Santam., Nova Hedwig. 82: 358 (2006)**

• *Myllaena elongata* (Matthews, 1838) (Coleoptera, Staphylinidae) Nl

**99. *Monoicomyces nigrescens* Thaxt., Proc. Am. Acad. Arts Sci. 35: 412 (1900)**

• *Atheta atramentaria* (Gyllenhal, 1810) (Coleoptera, Staphylinidae) Nl

• *Atheta* sp. Be

• *Brundinia marina* (Mulsant & Rey, 1853) (Coleoptera, Staphylinidae) Be^a^

• *Dilacra luteipes* (Erichson, 1837) (Coleoptera, Staphylinidae) Nl^b^

^a^ Host as Atheta (Actophylla) marina (Mulsant & Rey, 1853) in De Kesel et al. (2020).

^b^ Host as *Atheta luteipes* (Erichson, 1837) in Middelhoek (1943a).

**100. *Peyritschiella biformis* (Thaxt.) I.I. Tav., Mycol. Mem. 9: 270 (1985)**

• *Philonthus umbratilis* (Gravenhorst, 1802) (Coleoptera, Staphylinidae) Be

**101. *Peyritschiella dubia* (Thaxt.) I.I. Tav., Mycol. Mem. 9: 270 (1985)**

• *Philonthus politus* (Linnaeus, 1758) (Coleoptera, Staphylinidae) Be

**102. *Peyritschiella furcifera* (Thaxt.) I.I. Tav., Mycol. Mem. 9: 270 (1985)**

• *Philonthus albipes* (Gravenhorst, 1802) (Coleoptera, Staphylinidae) Nl^a^

• *Philonthus rectangulus* Sharp, 1874 Nl^a^

^a^ Fungus as *Dichomyces furciferus* Thaxt. in Middelhoek (1943a).

**103. *Peyritschiella heinemanniana* De Kesel, Belg. J. Bot. 131: 177 (1999) [1998**]

• *Xantholinus longiventris* Heer, 1839 (Coleoptera, Staphylinidae) Be

**104. *Peyritschiella princeps* (Thaxt.) I.I. Tav., Mycol. Mem. 9: 270 (1985)**

• *Bisnius cephalotes* (Gravenhorst, 1802) (Coleoptera, Staphylinidae) Be, Nl^a^

• *Bisnius sordidus* (Gravenhorst, 1802) Be, Nl^b^

• *Bisnius subuliformis* (Gravenhorst, 1802) Nl

• *Philonthus politus* (Linnaeus, 1758) (Coleoptera, Staphylinidae) Be

• *Philonthus varians* (Paykull, 1789) Nl^c^

• *Philonthus* sp. Be

^a^ Host as *Philonthus cephalotes* (Gravenhorst, 1802), fungus as *Dichomyces vulgatus* Thaxt. in Middelhoek (1943a, 1947a).

^b^ Host as *Philonthus sordidus* (Gravenhorst, 1802), fungus as *Dichomyces princeps* Thaxt. in Middelhoek (1941, 1943a, 1943b, 1943c), fungus also as *Dichomyces vulgatus* Thaxt. (variety *sensu*Thaxter 1908: 252) in Middelhoek (1943a, 1943b).

^c^ Fungus as *Dichomyces princeps* Thaxt. in Middelhoek (1941).

**105. *Peyritschiella protea* Thaxt., Proc. Am. Acad. Arts Sci. 35: 427 (1900)**

• *Anotylus insecatus* Gravenhorst, 1806 (Coleoptera, Staphylinidae) Be

• *Anotylus rugosus* (Fabricius, 1775) Be, Nl^a^

• *Anotylus* sp. Be

• Staphylinidae sp. indet. (Coleoptera, Staphylinidae) Be

^a^ Host as *Oxytelus rugosus* (Fabricius, 1775) in Middelhoek (1943a, 1947a).

**106. *Peyritschiella vulgata* (Thaxt.) I.I. Tav., Mycol. Mem. 9: 271 (1985)**

• *Philonthus albipes* (Gravenhorst, 1802) (Coleoptera, Staphylinidae) Nl^a^

^a^ Fungus as *Dichomyces vulgatus* Thaxt. in Middelhoek (1943b, 1943c).

**107. *Phaulomyces simplocariae* De Kesel, Mycotaxon 50: 192 (1994)**

• *Simplocaria semistriata* Fabricius, 1794 (Coleoptera, Byrrhidae) Be

**108. *Rhachomyces canariensis* Thaxt., Proc. Am. Acad. Arts Sci. 35: 436 (1900)**

• *Trechus obtusus* Erichson, 1837 (Coleoptera, Carabidae) Be, Nl^a^

• *Trechus quadristriatus* (Schrank, 1781) Be

• *Trechus* sp. Be

^a^ New record: Noord-Holland Province, Zuid-Kennemerland National Park, 17 Oct. 2016, *leg.* M. Boeken, pitfall trap, on *Trechus obtusus* Erichson, 1837 (Coleoptera, Carabidae), slides D. Haelew. 1242a (GENT, 9 thalli, right margin of pronotum) and D. Haelew. 1242b (GENT, 3 juvenile thalli, elytra). *Ibid.*, 5 Jun. 2017, *leg.* M. Boeken, pitfall trap, on *Trechus obtusus*, slide D. Haelew. 1388a (GENT, 1 submature thallus, tip of left elytron).

**109. *Rhachomyces furcatus* (Thaxt.) Thaxt., Proc. Am. Acad. Arts Sci. 30: 467 (1895) [1894**]

• *Othius punctulatus* (Goeze, 1777) (Coleoptera, Staphylinidae) Be

• *Othius subuliformis* Stephens, 1833 Be^a^, Nl

^a^ Host as *Othius myrmecophilus* Kiesenwetter, 1843 in De Kesel et al. (2020).

**110. *Rhachomyces lasiophorus* (Thaxt.) Thaxt., Proc. Am. Acad. Arts Sci. 30: 468 (1895) [1894**]

• *Acupalpus dubius* Schilsky, 1888 (Coleoptera, Carabidae) Be

• *Acupalpus exiguus* Dejean, 1829 Be, Nl

• *Anthracus consputus* (Duftschmid, 1812) (Coleoptera, Carabidae) Nl

**111. *Rhachomyces philonthinus* Thaxt., Proc. Am. Acad. Arts Sci. 35: 435 (1900)**

• *Bisnius fimetarius* (Gravenhorst, 1802) (Coleoptera, Staphylinidae) Be, Nl^a^

• *Philonthus cruentatus* (Gmelin, 1790) (Coleoptera, Staphylinidae) Nl^b^

• *Philonthus fumarius* (Gravenhorst, 1806) Be

• *Philonthus marginatus* (Müller, 1764) Be, Nl

• *Philonthus rectangulus* Sharp, 1874 Be

• *Philonthus varians* (Paykull, 1789) Be, Nl^b^

• *Philonthus* sp. Be

^a^ Host as *Philonthus fimetarius* (Gravenhorst, 1802) in Middelhoek (1943a, 1943d).

^b^ Fungus as *Rhachomyces* ‘*philonthi*’ Thaxt. in Middelhoek (1943b).

**112. *Rhachomyces pilosellus* (C.P. Robin) Thaxt., Proc. Am. Acad. Arts Sci. 30: 467 (1895) [1894**]

• *Lathrobium fulvipenne* (Gravenhorst, 1806) (Coleoptera, Staphylinidae) Be

• *Lathrobium geminum* Kraatz, 1857 Be

**113. *Rhachomyces spinosu* s Santam. & A.D. Cuesta-Segura, Nova Hedwig. 110: 362 (2020)**

• *Syntomus foveatus* (Geoffroy, 1785) (Coleoptera, Carabidae) Be^a^

^a^ Fungus as *Rhachomyces sciakyi* W. Rossi in De Kesel et al. (2020)

**114. *Rhachomyces tenenbaumii* J. Siemaszko & Siemaszko, Polsk. Pism. Entomol. 6: 205 (1928)**

• *Thalassophilus longicornis* (Sturm, 1825) (Coleoptera, Carabidae) Be

**115. *Rhadinomyces cristatus* Thaxt., Proc. Am. Acad. Arts Sci. 28: 180 (1893)**

• *Lathrobium brunnipes* (Fabricius, 1793) (Coleoptera, Staphylinidae) Be

• *Lathrobium castaneipenne* Kolenati, 1846 Be

• *Lathrobium elongatum* (Linnaeus, 1767) Be

• *Lathrobium fulvipenne* (Gravenhorst, 1806) Be

• *Lathrobium geminum* Kraatz, 1857 Be

• *Lathrobium* sp. Be

**116. *Rhadinomyces pallidus* Thaxt., Proc. Am. Acad. Arts Sci. 28: 180 (1893)**

• *Lathrobium elongatum* (Linnaeus, 1767) (Coleoptera, Staphylinidae) Nl

**117. *Rhynchophoromyces anacaenae* Scheloske, Parasitol. Schriftenr. 19: 143 (1969)**

• *Anacaena lutescens* (Stephens, 1829) (Coleoptera, Hydrophilidae) Be

**118. *Rickia dendroiuli* W. Rossi, Rev. Mycol. 41: 531 (1977)**

• Julida sp. indet. Be

**119. *Rickia laboulbenioides* De Kesel, Sterbeeckia 32: 6 (2013)**

• *Cylindroiulus latestriatus* (Curtis, 1845) (Julida, Julidae) Be, Nl

• *Cylindroiulus punctatus* Leach, 1814 Be

• Julida sp. indet. Be

**120. *Rickia peyerimhoffii* Maire, Bull. Sci. Fr. Belg. 7: 290 (1916)**

• *Scaphisoma* sp. (Coleoptera, Staphylinidae) Be

**121. *Rickia proteini* T. Majewski, Acta Mycol. 19: 191 (1985)**

• *Proteinus* sp. (Coleoptera, Staphylinidae) Be

**122. *Rickia wasmannii* Cavara, Malpighia 13: 182 (1899)**

• *Myrmica ruginodis* Nylander, 1846 (Hymenoptera, Formicidae) Nl

• *Myrmica sabuleti* Meinert, 1861 (Hymenoptera, Formicidae) Be, Nl^a^

• *Myrmica scabrinodis* Nylander, 1846 Nl

^a^ Host as *Myrmica scabrinodis* Nylander, 1846 in Haelewaters (2012).

**123. *Siemaszkoa fennica* Huldén, Karstenia 23: 63 (1983)**

• *Ptenidium formicetorum* Kraatz, 1851 (Coleoptera, Ptiliidae) Nl

**124. *Siemaszkoa ptenidii* (Scheloske) I.I. Tav. & T. Majewski, Mycotaxon 3: 204 (1976)**

• *Ptenidium* sp. (Coleoptera, Ptiliidae) Be

**125. *Stichomyces conosomatis* Thaxt., Proc. Am. Acad. Arts Sci. 37: 38 (1901)**

• *Sepedophilus marshami* (Stephens, 1832) (Coleoptera, Staphylinidae) Be

• *Sepedophilus nigripennis* (Stephens, 1832) Be, Nl

• *Sepedophilus pedicularius* (Gravenhorst, 1802) Be

• *Sepedophilus testaceus* (Fabricius, 1792) Nl

• *Sepedophilus* sp. Be

**126. *Stigmatomyces baeri* H. Karst., Chemismus Pfl.-Zelle: 78 (1869)**

• “*Fannia canicularis*” (Linnaeus, 1761) (Diptera, Fanniidae) Nl^a^

^a^ Host as *Homalomyia canicularis* (Linnaeus, 1761) in Boedijn (1923). The host identification may have been incorrect; *Fannia canicularis* is typically associated with *Fanniomyces ceratophorus* Whisler, whereas *S. baeri* is typically found on *Musca domestica* Linnaeus, 1758 (Diptera, Muscidae).

**127. *Stigmatomyces crassicollis* Thaxt., Proc. Am. Acad. Arts Sci. 52: 661 (1917)**

• *Leptocera caenosa* (Rondani, 1880) (Diptera, Sphaeroceridae) Be

• *Leptocera fontinalis* (Fallén, 1826) Be

• *Leptocera lutosoidea* (Duda, 1938) Be

• *Opacifrons humida* (Haliday, 1836) (Diptera, Sphaeroceridae) Be

• *Spelobia rufilabris* (Stenhammar, 1855) (Diptera, Sphaeroceridae) Be

• Sphaeroceridae sp. indet. (Diptera) Be

**128. *Stigmatomyces divergatus* Thaxt., Mem. Am. Acad. Arts Sci. 16: 122 (1931)**

• *Apteromyia claviventris* (Strobl, 1909) (Diptera, Sphaeroceridae) Be

• *Spelobia parapusio* (Dahl, 1909) (Diptera, Sphaeroceridae) Be

• *Spelobia* sp. Be

**129. *Stigmatomyces entomophilus* (Peck) Thaxt., Proc. Am. Acad. Arts Sci. 36: 398 (1900) [1901**]

• *Drosophila funebris* (Fabricius, 1787) (Diptera, Drosophilidae) Nl

**130. *Stigmatomyces hydrelliae* Thaxt., Proc. Am. Acad. Arts Sci. 36: 404 (1900) [1901**]

• *Hydrellia albilabris* (Meigen, 1830) (Diptera, Ephydridae) Nl^a^

^a^ New record: Noord-Brabant Province, Udenhout, nature reserve De Brand, 51.631777N 5.132998E, 14–21 Jun. 1990, *leg.* Working Group Insects of the Royal Dutch Natural History Association (KNNV), malaise trap (van Zuijlen et al. 1996), on *Hydrellia albilabris* (Meigen, 1830) (Diptera, Ephydridae), slide WR1746 (will be deposited at FI, Herbarium Universitatis Florentinae, Florence, Italy), *det.* W. Rossi, *comm.* J.W.A. van Zuijlen.

**131. *Stigmatomyces limosinae* Thaxt., Proc. Am. Acad. Arts Sci. 36: 406 (1900) [1901**]

• *Spelobia clunipes* (Meigen, 1830) (Diptera, Sphaeroceridae) Be

• *Spelobia talparum* (Richards, 1927) Nl

**132. *Stigmatomyces majewskii* H.L. Dainat, Manier & Balazuc, Bull. Trimest. Soc. Mycol. Fr. 90: 171 (1974)**

• *Drosophila subobscura* Collin, 1936 (Diptera, Drosophilidae) Nl

**133. *Stigmatomyces minilimosinae* T. Majewski, Polish Bot. Stud. 1: 122 (1990)**

• *Minilimosina parvula* (Stenhammar, 1855) (Diptera, Sphaeroceridae) Be

**134. *Stigmatomyces platensis* Speg., Anal. Mus. Nac. Hist. Nat. B. Aires 29: 676 (1917)**

• *Paralimosina fucata* (Rondani, 1880) (Diptera, Sphaeroceridae) Be

• *Paralimosina subcribrata* (Rohacek, 1977) Be

**135. *Symplectromyces vulgaris* (Thaxt.) Thaxt., Mem. Am. Acad. Arts Sci. 13: 315 (1908)**

• *Philonthus* sp. (Coleoptera, Staphylinidae) Be

• *Quedius curtipennis* Bernhauer, 1908 (Coleoptera, Staphylinidae) Be

• *Quedius fuliginosus* (Gravenhorst, 1802) Be

• *Quedius fumatus* (Stephens, 1833) Be

• *Quedius lateralis* (Gravenhorst, 1802) Nl

• *Quedius levicollis* (Brullé, 1832) Be^a^

• *Quedius maurorufus* (Gravenhorst, 1806) Nl

• *Quedius mesomelinus* (Marsham, 1802) Be, Nl

• *Quedius* sp. Be

^a^ Host as *Quedius tristis* (Gravenhorst, 1802) in De Kesel et al. (2020).

**136. *Teratomyces actobii* Thaxt. Proc. Am. Acad. Arts Sci. 29: 98 (1894)**

• *Gabrius nigritulus* (Gravenhorst, 1802) (Coleoptera, Staphylinidae) Be

• *Gabrius* sp. Be

**137. *Teratomyces philonthi* Thaxt., Proc. Am. Acad. Arts Sci. 35: 432 (1901)**

• *Gabrius nigritulus* (Gravenhorst, 1802) (Coleoptera, Staphylinidae) Be

• *Gabrius trossulus* (Nordmann, 1837) Nl^a^

• *Gabrius* sp. Be

• *Quedius nitipennis* (Stephens, 1833) (Coleoptera, Staphylinidae) Be

• *Quedius* sp. Be

^a^ Host as *Philonthus trossulus* Nordmann, 1837 in Middelhoek (1943a).

**138. *Troglomyces manfrediae* S. Colla [as ‘manfredii’], Nuovo G. Bot. Ital. 39: 451 (1932)**

• Julida sp. indet. Be

**139. *Troglomyces triandrus* Santam. & Enghoff, Organ. Divers. Evol. 15: 253 (2015)**

• *Archiboreoiulus pallidus* (Brade-Birks, 1920) (Julida, Blaniulidae) Be

**140. *Zodiomyces vorticellarius* Thaxt., Proc. Am. Acad. Arts Sci. 25: 263 (1891)**

• *Helochares punctatus* (Sharp, 1869) (Coleoptera, Hydrophilidae) Nl

• *Helochares* sp. Be

### Doubtful records and combinations

*Laboulbenia elegans* Thaxt. on *Harpalus affinis* (Schrank, 1781) (Coleoptera, Carabidae) [as *Harpalus aeneus* (Fabricius, 1775)] (Middelhoek 1949). This material could not be verified since the Middelhoek collection is currently untraceable, but it likely represents *L. coneglianensis*. *Laboulbenia coneglianensis* is reported from species of *Harpalus* Latreille, 1802 and *Ophonus* Dejean, 1821 in Europe, whereas *L. elegans* is thus far only confirmed from New England, USA (Thaxter 1890, 1896).

*Laboulbenia flagellata* [as *Laboulbenia elongata* Thaxt.] on *Calathus erratus* (Sahlberg, 1827) (Coleoptera, Carabidae) (Middelhoek 1947b). The material is incomplete and impossible to verify. Given the host, it is doubtful that this report represents *L. flagellata*. Possibly it is *L. calathi* T. Majewski, which is already known from the Netherlands (Haelewaters et al. 2012b).

*Laboulbenia flagellata* on *Pterostichus nigrita* (Paykull, 1790) (Coleoptera, Carabidae) (Meijer 1975). This record possibly represents *L. pseudomasei* Thaxt. but we cannot verify because the material of Meijer is untraceable. *Pterostichus nigrita* is routinely reported as host to *L. pseudomasei*, not *L. flagellata* (Thaxter 1899; Scheloske 1969; Majewski 1994; Santamaría 1998; De Kesel et al. 2020). Both species are easily distinguished with morphological characters.

*Laboulbenia pedicellata* on *Trechus quadristriatus* (Schrank, 1781) (Coleoptera, Carabidae) (Meijer 1975). This would be the only worldwide record of *L. pedicellata* on a species of *Trechus* Clairville, 1806 and thus is likely incorrect. *Laboulbenia pedicellata* is generally reported on species of *Bembidion* Latreille, 1802 sensu lato (Coleoptera, Carabidae) and *Dyschirius* Bonelli, 1810 (Coleoptera, Carabidae) (Haelewaters et al. 2019a).

## Discussion

### New species and new records

In this paper, we describe two new species of Laboulbeniales based on the combination of molecular data, morphology, and ecology (host association). These are *Hesperomyces halyziae* on *Halyzia sedecimguttata* in Belgium and the Netherlands, and *Laboulbenia quarantenae* on *Bembidion biguttatum* in Belgium. Additionally, *Laboulbenia aubryi* and *Rhachomyces spinosus* are newly reported from Belgium. Seven previously described species of Laboulbeniales are reported for the first time from the Netherlands: *Chitonomyces melanurus*, *Euphoriomyces agathidii*, *Laboulbenia fasciculata*, *Laboulbenia metableti*, *Laboulbenia pseudomasei*, *Rhachomyces canariensis*, and *Stigmatomyces hydrelliae*.

The report of *L. aubryi* from Belgium is only the third one from Europe. *Laboulbenia aubryi* was thus far only recorded from India, Nepal, Poland, and Spain (type). Reported hosts are species in *Amara* Bonelli, 1810 (= *Bradytus* Stephens, 1827, = *Leironotus* Ganglbauer, 1892) (Santamaría et al. 1991; Santamaría 1998; Majewski 1999), a diverse genus that is only exceptionally reported with Laboulbeniales (Santamaría et al. 1991). Scheloske (1969) mentioned *L. flagellata* on *Amara plebeja* (Gyllenhal, 1810), but considered it an accidental host (“Zufallswirt”). Moreover, based on its simple outer appendage, *L. aubryi* can easily be separated from *L. flagellata*. The closest related species, morphologically speaking, is *L. argutoris* Cépède & F. Picard, but *L. aubryi* can be separated from it by the insertion cell that is free from the perithecium wall and by the structure of its inner appendage (Santamaría 1998).

*Rhachomyces spinosus* was recently described from Spain (Santamaría et al. 2020). The most characteristic feature of this species is the spinous process on the second cell of the primary appendage, absent in similar species *R. lavagnei* (F. Picard) W. Rossi and *R. sciakyi* W. Rossi. The reported host for *R. spinosus* in both Belgium and Spain is *Syntomus foveatus* (Coleoptera, Carabidae). *Rhachomyces lavagnei* is found on *Microlestes* spp. and *R. sciakyi* on *Pseudomesolestes* sp. All these hosts are placed in the subtribe Dromiusina (Harpalinae, Lebiini); it is possible that these species of *Rhachomyces* have a high degree of host specificity, which will only come to light as more material will be collected.

*Chitonomyces melanurus* is easily recognized from other congeneric species by the apically hooked, dark brown to blackish basal cell of its primary appendage. Nine species of *Chitonomyces* Peyr. occur in Europe, all of them occupying a specific position of the host integument. *Chitonomyces melanurus* grows almost exclusively on the upper margin of the left elytron of *Laccophilus* Leach, 1815 water beetles (Coleoptera, Dytiscidae). It has thus far has been reported in Europe from Austria (type), Belgium, Croatia, Finland, France, Germany, Hungary, Italy, Poland, Spain, Ukraine, United Kingdom; also found in Asia and Africa (Bánhegyi 1960; Huldén 1983; Santamaría et al. 1991; Majewski 1994; De Kesel and Werbrouck 2008; Rossi 2018).

The Dutch report of *E. agathidii* is found on *Agathidium laevigatum*, the host species from which the type was described (Maire 1920). *Euphoriomyces agathidii* is thus far found on members of *Agathidium* Panzer, 1796, *Amphicyllis* Erichson, 1845, and *Cyrtusa* Erichson, 1842 (Coleoptera, Leiodidae) in Bulgaria, Germany, Italy, Morocco (type), Poland, South Korea, Spain, and Sweden (Huldén 1983; Majewski 1994; Lee et al. 2007; Rossi et al. 2018). Our material is consistent with *E. agathidii*, with two mature perithecia at one side and a third, immature perithecium at the other side of the receptacular axis.

*Laboulbenia fasciculata* is recognized by the receptacular cell V, which proliferates upwards in a series of 4–8 superposed cells V’ gradually decreasing in size. Each of these cells V’ gives rise to a small trapezoidal cell that carries an appendage consisting of cells separated by dark and constricted septa. This species is very widespread, with reports across Europe, in Africa, Asia, and North and South America. Hosts are members of Carabidae, often *Chlaenius* Bonelli, 1810 (subfamily Harpalinae) and *Patrobus* Dejean, 1821 (subfamily Trechinae), but also several other genera in subfamilies Cicindelinae, Brachininae, Harpalinae, Nebriinae, Omophroninae, Patrobinae, and Trechinae (Santamaría et al. 1991). The reports on *Omophron* spp. are sometimes considered a form of *L. fasciculata* but this is not accepted by all (Spegazzini 1914; Majewski 1994; but Santamaría 1998).

The status of *L. metableti* as a separate species has been disputed. Formally synonymized with *L. notiophili* by Rossi and Santamaría (2006), De Kesel et al. (2020) reinstated *L. metableti* as a separate species based on characteristics of the appendage system. This species has a European distribution, with reports in Andorra, Austria, Belgium, Finland, Germany (type), Hungary, Italy, Poland, Russia, and the United Kingdom (reviewed in Rossi and Santamaría 2006). Hosts are species of *Syntomus* Hope, 1838 (= *Metabletus* Schmidt-Goebel, 1846) (Coleoptera, Carabidae, Harpalinae, Lebiini). We propose using molecular characters to resolve the debate given the taxonomic confusion of species of *Laboulbenia* on European hosts in the Lebiini tribe: *L. baetica* Balazuc, *L. blanchardii* Cépède, *L. cymindicola* Speg., *L. metableti*, *L. notiophili*, and *L. pulchella* Speg.

*Laboulbenia pseudomasei* is recognized by cell V that has an internal convex margin and is separated from the perithecium (Villarreal et al. 2010). Cell V sometimes proliferates into a simple or divided branch that grows upwards between the perithecium and insertion cell (Majewski 1994; Santamaría 1998). Rossi and Weir (1997) illustrated that *L. pseudomasei* can be morphologically highly variable even on a single host insect. Also in the newly reported material from the Netherlands, *L. pseudomasei* was variable, with the thallus from the right elytron without proliferation of cell V, and the thallus from the prosternum with proliferation of cell V. The geographic distribution of *L. pseudomasei* is problematic; many old records are unillustrated and the specimens are not preserved (Rossi and Weir 1997).

*Rhachomyces canariensis* was described from Tenerife (Thaxter 1900) and has since been reported from several countries in Europe and North Africa, Madeira, and the Canary Islands, always associated with species of *Trechus* Clairville, 1806 (Coleoptera, Carabidae) (Arndt and Santamaría 2004). Majewski (1994) noted the variability of this species and Tavares (1985) suggested material from large geographic distances to the type locality be segregated into a separate taxon.

The only species of Laboulbeniales found on *Hydrellia* Robineau-Desvoidy, 1830 flies (Diptera, Ephydridae) is *S. hydrelliae*. Thaxter (1901) described it from Kittery Point in Maine, USA (Thaxter 1901) and it has since then been reported in Finland, France, Italy, Poland, Portugal, Russia, the United Kingdom (Santamaría and Rossi 1993, Weir and Rossi 1995), and New Zealand (Hughes et al. 2004). The new report from the Netherlands is the first one on the European continent in 25 years. *Stigmatomyces hydrelliae* is recognized by its straight appendage with sterile basal cell and stout antheridia, the spiralled cell walls of the perithecium, and the rounded perithecial apex with one of the lip cells forming a slender, bluntly pointed projection. Hughes et al. (2004) noted that *S. hydrelliae* thalli from New Zealand are different in their perithecial wall cells not being spiralled and lacking apical projections at the perithecial apex.

### Checklist

The current list of thallus-forming Laboulbeniomycetes from Belgium and the Netherlands includes 140 species. Sixty-three species have been found in both countries. A total of 118 species are found in Belgium, and 85 species in the Netherlands. Of the 140 species in the checklist, 55 have not (yet) been reported from the Netherlands, and 22 species have not (yet) been reported from Belgium. Laboulbeniales research in both Belgium and the Netherlands has also resulted in the discovery of new taxa; over the years, 16 species have been described based on material from Belgium and/or the Netherlands (Table 3). It is remarkable that we keep finding undescribed species in two of the most urbanized countries in the world. The reason for this can be found in the fact that Laboulbeniomycetes are severely understudied; only a handful of researchers work on these fungi. In addition, some of the most recently described species are the result of previously unavailable molecular data, long-term study of humid habitats, and focus on unexplored niches.

**Table 3.** Species of Laboulbeniales described based on type material from Belgium (Be) and the Netherlands (Nl).

Country	Laboulbeniales species	Host species	Host classification	Reference
Nl	*Asaphomyces tubanticus* [as *Barbariella tubantica*]	*Catops nigricans*	Coleoptera, Leiodidae	Middelhoek (1949)
Nl	*Cantharomyces elongatus*	*Syntomium aeneum*	Coleoptera, Staphylinidae	Haelewaters and De Kesel (2013)
Nl	*Bordea denotata*	*Bibloporus bicolor*	Coleoptera, Staphylinidae	Haelewaters et al. (2014)
Be	*Cryptandromyces euplecti*	*Euplectus sanguineus*	Coleoptera, Staphylinidae	Santamaría (2001)
Be, NL	*Diphymyces kaaistoepi*	*Choleva cisteloides*, *C. fagniezi*	Coleoptera, Leiodidae	De Kesel and Haelewaters (2019)
Be, NL	*Hesperomyces halyziae*	*Halyzia sedecimguttata*	Coleoptera, Coccinellidae	This paper
Nl	*Laboulbenia barbara*	*Philonthus punctus*	Coleoptera, Staphylinidae	Middelhoek (1943a)
Be	*Laboulbenia quarantenae*	*Bembidion biguttatum*	Coleoptera, Carabidae	This paper
Be	*Laboulbenia elaphri*	*Elaphrus cupreus*	Coleoptera, Carabidae	Spegazzini (1915)
Be	*Laboulbenia hyalopoda*	*Paradromius linearis*	Coleoptera, Carabidae	De Kesel (1998)
Be, NL	*Laboulbenia littoralis*	*Cafius xantholoma*	Coleoptera, Staphylinidae	De Kesel and Haelewaters (2014)
Nl	*Mimeomyces zeelandicus*	*Heterothops binotatus*	Coleoptera, Staphylinidae	Middelhoek (1943a)
Be	*Peyritschiella heinemanniana*	*Xantholinus longiventris*	Coleoptera, Staphylinidae	De Kesel (1999)
Be	*Phaulomyces simplocariae*	*Simplocaria semistriata*	Coleoptera, Byrrhidae	De Kesel (1994)
Be, NL	*Rickia laboulbenioides*	*Cylindroiulus latestriatus*	Julida, Julidae	De Kesel et al. (2013)
Be	*Troglomyces triandrus*	*Archiboreoiulus pallidus*	Julida, Blaniulidae	Enghoff and Santamaría (2015)

This checklist is based on fungal records obtained from at least 283 host species (including only those identified to species level). To increase the number of thallus-forming Laboulbeniomycetes known from Belgium and the Netherlands, future research should focus on screening Acari (with *Rickia*), Blattodea (*Herpomyces*–especially in the Netherlands), Coleoptera (many genera), Corixidae (*Coreomyces*), Diplopoda (*Diplopodomyces*, *Troglomyces*), Diptera (*Stigmatomyces*), Hebridae (*Tavaresiella*, *Triceromyces*), and Mallophaga (*Trenomyces*). Within Coleoptera, the beetles, aquatic and semi-aquatic hosts, such as Dytiscidae (*Chitonomyces*), Hydraenidae (*Autoicomyces*, *Hydrophilomyces*, *Thripomyces*), and Hydrophilidae (*Chaetarthriomyces*, *Eusynaptomyces*) deserve special attention. More genera of Laboulbeniales that are currently not yet recorded from either Belgium or the Netherlands, could be discovered on Anthicidae (*Dioicomyces*), Ptiliidae (*Siemaszkoa*), Silvanidae (*Cucujomyces*), Staphylinidae (*Amorphomyces*, *Diplomyces*, *Dipodomyces*, *Haplomyces*, *Mimeomyces*, *Sphaleromyces*), and Tenebrionidae (*Dimeromyces*).

As Laboulbeniomycetes research progresses, lesser known host groups will need to be incorporated into our studies. This will eventually require intensified collaborations with specialist entomologists, as well as screening museum insect collections and the use of different collecting methods. That different sampling techniques have an impact on Laboulbeniales studies may be illustrated by our work with *Rickia wasmannii* Cavara. Based on pitfall trapping, Haelewaters et al. (2015a) reported *R. wasmannii* from three host species: *Myrmica sabuleti* Meinert, 1861 (parasite prevalence 38%), *M. scabrinodis* Nylander, 1846 (11%), and *M. ruginodis* Nylander, 1846 (0.55%). Direct sampling from a *M. scabrinodis* nest at the same locality in the Netherlands, however, resulted in a 100% prevalence (De Kesel et al. 2016).

Finally, undersampled habitats have been cited repeatedly as one of the main sources to find undescribed fungi (e.g., Blackwell 2011, Hawksworth and Lücking 2017, Wijayawardene et al. 2020). This is especially true for the Laboulbeniomycetes. Sampling from dung, fresh and brackish water, animal nests, caves, carcasses, and rotting plant debris has greatly contributed to discoveries in this field of research, not only adding to numbers of described species but also building on our understanding of the ecology of these minute fungi. For example, Pfliegler et al. (2016) sampled ants and their associates from ant nests and, for the first time since its description (Cavara 1899), *R. wasmannii* was observed on hosts other than *Myrmica*, including inquiline mites and a fly larva. A survey of Laboulbeniales from coprophilic beetles on Galloway dung in Belgium resulted in two reports of species that until then had only been found in Poland, thus representing a large geographical range expansion (De Kesel 2010). And signal crayfish traps in nature reserve ‘De Kaaistoep’ have thus far revealed an undescribed species of *Diphymyces* (De Kesel and Haelewaters 2019) and more material is awaiting detailed study.

### Key to Laboulbeniomycetes from Belgium and the Netherlands

**Table:** 

1	Dioecious; on Blattodea (cockroaches)	**50 (*Herpomyces*)**
–	Thalli mostly monoecious; on other host groups	**2**
2	Perithecial wall cells numerous, subequal, always ≥ 6 cells per vertical row	**3**
–	Perithecial wall with < 6 cells per vertical row	**7**
3	Receptacle uniseriate, composed of numerous superposed cells	**5**
–	Receptacle multiseriate, often massive	**4**
4	Receptacle turbinate, with apical depression holding numerous sterile appendages, stalked perithecia and antheridial branchlets; on Hydrophilidae	***Zodiomyces vorticellarius***
–	Receptacle not turbinate, bearing numerous perithecia and appendages laterally; on Carabidae and Staphylinidae	***Euzodiomyces lathrobii***
5	Perithecium with an apical, darkened rostrum; receptacle with 4–5 cells; on Ptiliidae	***Kainomyces rehmanii***
–	Perithecium without a rostrum; receptacle with > 5 cells	**6**
6	Perithecium long-necked, without lobes or fine appendages on the perithecial wall; on Hydrophilidae	***Rhynchophoromyces anacaenae***
–	Perithecium without long neck, ostiolum with 4 fine ligulae, lower wall bearing slender ramified appendices; on Dryopidae	***Helodiomyces elegans***
7	Antheridia simple, flask shaped; release of spermatia through small necks	**8**
–	Antheridia grouped into a compound structure with wall	**44**
8	Sterile appendages unicellular with black basal septum; antheridia small, always with black basal septum; receptacle formed by 3 vertical tiers of cells (not always clear), at least one tier partly or entirely flanking the perithecium	**52 (*Rickia*)**
–	Not this combination	**9**
9	Suprabasal cell of the receptacle (cell II) produces multi-celled secondary appendages; the latter supporting a perithecium (with cell VI) at their base	***Compsomyces lestevae***
–	Cell II not producing secondary appendages	**10**
10	Perithecial wall with an elongated accessory cell along its outer venter; unicellular outgrowths are formed above the foot; on *Cercyon* (Hydrophilidae)	**11**
–	Perithecium without accessory cell; no such outgrowths above the foot	**12**
11	Lower receptacular cells isodiametric; perithecium neck more or les straight	**Hydrophilomyces cf. gracilis**
–	Lower receptacular cells flattened; perithecium neck strongly curved	**Hydrophilomyces cf. hamatus**
12	Cell VII and basal cells of the perithecium clearly visible in mature perithecia	**13**
–	Cell VII and basal cells of the perithecium not visible in mature perithecia	**40**
13	Receptacle produces longitudinal septa, leading to a suprabasal complex with numerous secondary appendices	**14**
–	Receptacle stays a series of superposed cells, rarely forming longitudinal septa, not forming a suprabasal complex or secondary appendices	**20**
14	Receptacle composed of a series of superposed cells (4–5 or more), each forming on one side a basal cell with numerous, fairly large, pigmented and multicellular appendages; thalli usually with only one perithecium	**56 (*Rhachomyces*)**
–	Not with these features	**15**
15	Thallus hyaline; appendages not in bunches; On Cholevinae (Leiodidae)	***Asaphomyces tubanticus***
–	Thallus moderately to deeply pigmented in some parts; appendages appear in bunches on the receptacle	**16**
16	Receptacle asymmetrical	**17**
–	Receptacle mostly symmetrical	**18**
17	Antheridia in lateral series on fertile appendages; dorsal and ventral cell of the triangular receptacle supporting a series of appendages and their basal cells; perithecium stalked by elongated cells VI and VII	***Idiomyces peyritschii***
–	Antheridia never organized in lateral series; appendages not in series; receptacle 5-celled; cells VI and VII relatively short	**62 (*Laboulbenia*)**
18	Appendages with pointed-curved tips, darkened septa; antheridia terminal, flask shaped, not forming ramifications with age	**19 (*Teratomyces*)**
–	Appendages with rounded tips, with series of intercalary antheridia, the latter ramifying into new appendages with age	***Symplectromyces vulgaris***
19	Cells I and II from receptacle becoming brown with age; basal cells of appendages with laterally aligned antheridia/septa	***Teratomyces philonthi***
–	Cell I hyaline, contrasting with a deep blackened cell II; basal cells of appendages without such laterally aligned septa	***Teratomyces actobii***
20	Primary appendage bicellular, both cells separated by a dark constricted septum; antheridium below the primary appendage; on aquatic Coleoptera	**21**
–	Primary appendage more developed	**22**
21	All 4 vertical tiers of the perithecial wall have 4 cells each	**109 (*Chitonomyces*)**
–	Only 2 vertical tiers of the perithecial wall have 4 cells, the others have 6 cells; on Haliplidae	***Hydraeomyces halipli***
22	Receptacle composed of ≥ 4 cells	**23**
–	Receptacle composed of ≤ 3 cells	**26**
23	Primary receptacle composed of a chain of cells (≥ 3)	**24**
–	Primary receptacle composed of cells I and II, entire receptacle with five cells	**62 (*Laboulbenia*)**
24	Perithecium with obtuse apex and inconspicuous neck	***Misgomyces dyschirii***
–	Perithecium with long neck and differentiated venter	**25**
25	Antheridia sessile, develop as corner cells of the primary appendage; Receptacle cells flattened, broadening upwards	***Ecteinomyces trichopterophilus***
–	Antheridia not sessile but formed on lateral branchlets; receptacle cells elongate	***Botryandromyces heteroceri***
26	Cell III flattened and entirely appressed against the perithecium; on Julida	**113 (*Troglomyces*)**
–	Cell III different; on Hexapoda	**27**
27	On Coleoptera	**29**
–	Not on Coleoptera	**28**
28	Basal cell of appendage dark; perithecial apex with outgrowths; on *Forficula* (Dermaptera, Forficulidae)	***Distolomyces forficulae***
–	On Diptera	**114 (*Fanniomyces* & *Stigmatomyces*)**
29	Primary appendage easily breaking off at its strongly narrowed basal cell; on Kateretidae	***Aphanandromyces audisioi***
–	Primary appendage persistent	**30**
30	Receptacle cells (I, II, III) more or less superposed	**31**
–	Receptacle cells not superposed (cell I and III touching)	**39**
31	Distal cell of primary appendage is a simple antheridium, with efferent neck and spine	***Bordea denotata***
–	Primary appendage without such a single and terminal antheridium	**32**
32	Antheridial structures are born on corner cells of appendage axis cells	**33**
–	Antheridial branches not born from corner cells	**36**
33	Basal (m, n, n’) and stalk cells (VI, VII) of the perithecium small (together < 25 µm long); on Hydrophilidae	***Chaetarthriomyces crassiappendicatus***
–	Basal (m, n, n’) and stalk cells (VI, VII) ≥ 25 µm long; on Staphylinidae	**34**
34	Cell III mostly without antheridial branches, with or without perithecium; cells VI and VII of similar length	***Stichomyces conosomatis***
–	Cell III always with antheridial branches, never with perithecium; cell VI much taller than cell VII	**35**
35	Thallus forms perithecia and corner cells on one side (anterior)	***Rhadinomyces pallidus***
–	Thallus forms perithecia and corner cells on both sides (anterior and posterior)	***Rhadinomyces cristatus***
36	Primary appendage simple, composed of numerous similar superposed cells	**124 (*Cryptandromyces*)**
–	Primary appendage branched	**37**
37	Cell VI adnate to cell II; exclusively on Cholevinae (Leiodidae)	***Diphymyces kaaistoepi***
–	Cell VI supported by cell II; mostly on Staphylinidae, rarely on Cholevinae (Leiodidae)	**38**
38	Cell I tall and elongated, cell II flattened	***Mimeomyces zeelandicus***
–	Cell I very short, cell II not flattened (isodiametric)	**126 (*Corethromyces*)**
39	Perithecial tip with prominent ostiolar lips and lobes; appendage short, with sessile lateral antheridia on each cell; fresh thalli often greenish-yellow; on Coccinellidae	**127 (*Hesperomyces*)**
–	Perithecial tip without such lobes; appendage long, with lateral antheridia on few cells; not on Coccinellidae	**124 (*Cryptandromyces*)**
40	Receptacle between foot and cell VI with ≥ 3 cells.	**41**
–	Receptacle between foot and cell VI with 2 cells; foot entirely black	***Phaulomyces simplocariae***
41	Receptacle a series of superposed cells, many of which laterally producing perithecia and/or appendages	***Euphoriomyces agathidii***
–	Receptacle a series of superposed cells without lateral cells bearing perithecia and appendages	**42**
42	Receptacle with flattened and finely appendiculate cells above cell III; Foot entirely black; on Corixidae (Hemiptera)	***Coreomyces arcuatus***
–	Receptacle without such flattened cells above cell III; foot with a small blackish dot; on Ptiliidae	**43**
43	The appendage is a prolongation of the receptacle axis, the perithecium is lateral	***Siemaszkoa ptenidii***
–	The perithecium is often terminal and in continuation with the receptacular axis	***Siemaszkoa fennica***
44	Cell I laterally extending and supporting a series of cells derived from cell II; thallus dioecious	***Dimorphomyces myrmedoniae***
–	Cell I not laterally extending; thallus monoecious	**45**
45	Primary receptacle composed of a chain of ≥ 3 cells	***Misgomyces dyschirii***
–	Primary receptacle not a chain of cells	**46**
46	Primary appendage fertile, with a compound antheridium	**47**
–	Primary appendage sterile or absent	**49**
47	Compound antheridium with efferent neck and tall cell on the outer side; on Carabidae	***Eucantharomyces stammeri***
–	Compound antheridium different, never with efferent neck; on Staphylinidae and Dryopidae (= Parnidae)	**48**
48	Primary appendage is entirely transformed into a compound antheridium, with spine	***Haplomyces texanus***
–	Compound antheridium is an intercalary structure of the primary appendage	**129 (*Cantharomyces*)**
49	Receptacle formed by 3 horizontal tiers of cells; antheridia compound, sessile, often on the median series; sterile appendages unicellular	**134 (*Peyritschiella*)**
–	Receptacle differently organized; sterile appendages multicellular; antheridial structure stalked, large	**140 (*Monoicomyces*)**
50	Secondary receptacle (female thallus) without concentrically organized cells	***Herpomyces ectobiae***
–	Secondary receptacle a series of concentrically organized and flattened cells	**51**
51	Secondary receptacle ≤ 80 µm long, with rounded apex and partially darkened cells	***Herpomyces stylopygae***
–	Secondary receptacle ≥ 100 µm long, with pointed apex and without dark pigmentations	***Herpomyces periplanetae***
52	Perithecium almost entirely embedded in the receptacle	***Rickia peyerimhoffii***
–	Anterior part of the perithecium free	**53**
53	On Diplopoda	**54**
–	On other arthropods	**55**
54	Dorsal margin of the perithecium free in its upper third; anterior series of receptacle consisting of 2(–3) cells	***Rickia laboulbenioides***
–	Dorsal margin of the perithecium only free at the apex; Anterior series of receptacle consisting of > 2 cells	***Rickia dendroiuli***
55	Cell I 12–18 µm long; on Staphylinidae	***Rickia proteini***
–	Cell I 60–90 µm long; on *Myrmica* (Hymenoptera, Formicidae)	***Rickia wasmannii***
56	Primary appendage hyaline, 3-celled, different from other appendages; On *Syntomus* (Carabidae)	***Rhachomyces spinosus***
–	Primary appendage pigmented, identical to secondary appendages	**57**
57	Receptaculum between cells I and VI usually with < 6 cells, sterile appendages very long; on *Lathrobium* (Staphylinidae)	***Rhachomyces pilosellus***
–	Receptaculum between cells I and VI composed of ≥ 6 cells; sterile appendages do not exceed perithecial apex	**58**
58	Cells of the B-appendages of unequal length	**59**
–	Cells of the B-appendages of similar to equal length	**60**
59	B-appendages elongate, slender, tapering upwards; On *Philonthus* (Staphylinidae)	***Rhachomyces philonthinus***
–	B-appendages short, stout, width broad rounded apex; On *Thalassophilus* (Carabidae)	***Rhachomyces tenenbaumii***
60	Cell VI elongate and situated in the median to subapical part of the (secondary) receptacle; On *Othius* (Staphylinidae)	***Rhachomyces furcatus***
–	Cell VI short, distally on the (secondary) receptacle; on Carabidae	**61**
61	Perithecial apex with black spots; terminal cell of the B-appendages widest in the middle; on *Acupalpus* (Carabidae)	***Rhachomyces lasiophorus***
–	Perithecial apex hyaline; terminal cell of B-appendages cylindrical, usually proliferating; on *Trechus* (Carabidae)	***Rhachomyces canariensis***
62	Insertion cell absent	**63**
–	Insertion cell present	**65**
63	Appendages with large basal cells and dark septa; on Carabidae	***Laboulbenia fasciculata***
–	Appendages filiform, with fine basal cells and dark septal on Gyrinidae	**64**
64	Perithecium with two hyaline apical outgrowths, one straight one hooked	***Laboulbenia gyrinicola***
–	Both perithecial outgrowths with black spots, irregularly shaped	***Laboulbenia fennica***
65	On Carabidae	**66**
–	On Staphylinidae	**103**
66	Insertion cell free	**67**
–	Insertion cell attached to the posterior margin of the perithecium (not free)	**72**
67	Foot almost hyaline with only a small black dot	***Laboulbenia hyalopoda***
–	Foot entirely black	**68**
68	Cell V as tall as cell IV	***Laboulbenia clivinalis***
–	Cell V smaller than cell IV	**69**
69	Outer appendage not branched	**70**
–	Outer appendage branched	***Laboulbenia pseudomasei***
70	Inner appendage hardly branched, with a single antheridium	***Laboulbenia lecoareri***
–	Inner appendage branched, with multiple antheridia	**71**
71	Lower 4–5 cells of outer appendage deeply pigmented in their middle; ostiolar papillae not conspicuous; on *Syntomus* (Carabidae)	***Laboulbenia metableti***
–	Lower 4–5 cells of outer appendage evenly pigmented; ostiolar papillae conspicuous; on *Amara* (Carabidae)	***Laboulbenia aubryi***
72	Cell V as tall as cell IV, or almost so	**73**
–	Cell V smaller than cell IV	**78**
73	Outer wall of perithecium with knobs	***Laboulbenia egens***
–	Outer wall of the perithecium without knobs	**74**
74	Outer appendage without dark septum, growing beyond the perithecium	***Laboulbenia ophoni***
–	Outer appendage with at least one dark septum, not growing beyond the perithecium	**75**
75	Cells IV and V flattened, broader than long; On *Cillenus* (Carabidae)	***Laboulbenia lichtensteinii***
–	Cells IV and V isodiametric or longer than broad	**76**
76	Thallus and receptaculum poorly pigmented (yellow-amber); basal cell of outer appendage inflated; on *Pogonus* (Staphylinidae)	***Laboulbenia slackensis***
–	Thallus and receptaculum strongly pigmented; basal cell of outer appendage not so inflated	**77**
77	Cell III flattened and oblique; posterior margin of cell IV longer than the one from cell III; insertion cell extremely flat and opaque	***Laboulbenia luxurians***
–	Cell III not flattened; posterior margin of cell IV equal or shorter than the one from cell III; insertion cell well-formed and black	***Laboulbenia pedicellata***
78	Outer appendage not growing beyond the perithecium	***Laboulbenia murmanica***
–	Outer appendage growing beyond the perithecium	**79**
79	Outer appendage branched	**80**
–	Outer appendage not branched	**90**
80	Cell IV very long, often with a conspicuous dorso-apical bump, sometimes divided	**81**
–	Cell IV not so long, never divided, without dorso-apical bump	**82**
81	Outer appendage with > 2 branches; on *Stenolophus* (Carabidae)	***Laboulbenia anoplogenii***
–	Outer appendage consisting of 2 branches; on *Acupalpus* (Carabidae)	***Laboulbenia acupalpi***
82	Insertion cell on or above the middle of the perithecium; inner appendage less developed than outer appendage	**83**
–	Insertion cell below the middle of the perithecium	**84**
83	Thallus and receptaculum pale; septa from basal cells of outer appendage not darkened; on *Paranchus albipes* (Carabidae)	***Laboulbenia collae***
–	Thallus and receptaculum strongly pigmented; septa from basal cells of outer appendage darkened	***Laboulbenia vulgaris***
84	Outer side of the base of outer appendage strongly darkened	**85**
–	Outer side of the base of outer appendage not or only very slightly darkened	**87**
85	Thallus pale brown; appendages numerous, with tapering and pointed apices; on *Dicheirotrichus* (Carabidae)	***Laboulbenia giardii***
–	Thallus deep brown; appendages not so numerous, not tapering, with rounded apices	**86**
86	Septum II/III clearly shorter than septum II/VI; cell V clearer than surrounding structures; on *Harpalus* and *Ophonus* (Carabidae)	***Laboulbenia coneglianensis***
–	Septum II/III nearly as long as septum II/VI; cell V not much paler than surrounding structures; on *Brachinus* (Carabidae)	***Laboulbenia rougetii***
87	Thallus often bent, anterior side of the thallus concave	**89**
–	Thallus not so bent, anterior side of the thallus fairly straight	**88**
88	Insertion cell near the base of the perithecium; outer appendage often composed of 4–6(–8) branches, resulting from successive dichotomies starting at the suprabasal cell	***Laboulbenia quarantenae***
–	Insertion cell not so deep; outer appendage branched once or twice, not as dichotomies	***Laboulbenia flagellata* sensu lato**
89	Cell V quite small, less than half the length of cell IV; perithecium very slender, subcylindrical (not a stable feature); on *Harpalus* (Carabidae)	***Laboulbenia coneglianensis***
–	Cell V longer, usually more than half the length of cell IV; perithecium more ovate; on *Elaphrus* (Carabidae)	***Laboulbenia elaphri***
90	Insertion cell located at or above the middle of the perithecium; adaxial side of the perithecium half free	**91**
–	Insertion cell located well below the middle of the perithecium; adaxial side of the perithecium more than half free	**95**
91	Basal cells of outer appendage with darkened septa	**93**
–	Basal cells of outer appendage with normal septa	**92**
92	Cell VI broader than long	***Laboulbenia benjaminii***
–	Cell VI longer than broad	***Laboulbenia argutoris***
93	Basal cell of outer appendage inflated; inner appendage growing beyond the perithecium	***Laboulbenia inflata***
–	Basal cell of outer appendage normal; inner appendage never beyond perithecium	**94**
94	Inner appendage composed of a single antheridium supported by one basal cell; on *Asaphidion* (Carabidae)	***Laboulbenia thaxteri***
–	Inner appendage with ≥ 2 cells each supporting one or more antheridia	***Laboulbenia vulgaris***
95	Outer appendage rotated relative to the perithecium; on *Pterostichus diligens* (Carabidae)	***Laboulbenia kajanensis***
–	Outer appendage not rotated	**96**
96	Inner appendage growing far beyond the perithecium	**97**
–	Inner appendage not or hardly beyond the perithecium	**98**
97	Cell V clearly paler than surrounding cells and perithecium (III, IV and VI)	**98**
–	Cell V not paler than its surrounding cells	***Laboulbenia leisti***
98	Cell IV (and cell III) evenly and deeply pigmented	**100**
–	Cell IV (and cell III) hyaline or pigmented, their outer margins distinctly more pigmented than inner margins	**99**
99	Cell VI longer than broad; thallus ≥ 230 µm long; on *Calathus* (Carabidae)	***Laboulbenia calathi***
–	Cell VI isodiametric; thallus smaller; on *Demetrias*, *Notiophilus* and *Paradromius* (Carabidae)	***Laboulbenia notiophili* sensu lato**
100	Inner appendage hardly branched, with a single antheridium; cell IV longer than broad	***Laboulbenia lecoareri***
–	Inner appendage branched, with multiple antheridia; cell IV isodiametrical	**101**
101	Cell V minute; upper margin of cell IV 4–6× the width of cell V	**102**
–	Cell V larger; upper margin of cell IV only 1–2× the width of cell V	***Laboulbenia eubradycelli***
102	Lower 4–5 cells of outer appendage deeply pigmented in their middle; lower 3–4 cells of both branches of the inner appendage each producing a short straight branch	***Laboulbenia metableti***
–	Lower 4–5 cells of outer appendage evenly pigmented; inner appendage differently constructed	***Laboulbenia notiophili* sensu lato**
103	Cell V as long as cell IV, or almost so	**106**
–	Cell V smaller than cell IV	**104**
104	Insertion cell free from the perithecium; outer appendage branched	***Laboulbenia dubia***
–	Insertion cell attached to the posterior margin of the perithecium (not free); outer appendage not branched	**105**
105	Outer appendage with dark septa between basal cells; insertion cell near the base of the perithecium	***Laboulbenia stilicicola***
–	Outer appendage without dark septa at the basal cells; insertion cell near the middle of the perithecium	***Laboulbenia atlantica***
106	Outer appendage with at least one dark septum at the basal and suprabasal cells	**108**
–	Outer appendage without dark septa at the basal cells	**107**
107	Outer appendage forming a tuft of branches, posterior margins of both its basal and suprabasal cell entirely darkened	***Laboulbenia barbara***
–	Outer appendage simple or forked once; posterior margin of suprabasal cell of outer appendage with black remains of primary appendage	***Laboulbenia cristata***
108	Cell II hyaline; one black septum above the basal cell of the outer appendage	***Laboulbenia littoralis***
–	Anterior part of cell II pigmented black; black septa between all basal cells of inner and outer appendage	***Laboulbenia philonthi***
109	Perithecium with conspicuous outgrowths (spikes, thorns)	**110**
–	Perithecium without outgrowths; receptacle with outgrowth	**111**
110	Perithecial outgrowth, arising from the apical-most wall cell	***Chitonomyces paradoxus***
–	Perithecial outgrowth lateral, arising from sub-apical wall cell	***Chitonomyces aculeifer***
111	Suprabasal cell of the receptacle (Ia) flattened	***Chitonomyces bidessarius***
–	Suprabasal cell of the receptacle (Ia) isodiametric	**112**
112	Receptacular outgrowth hyaline, straight or arcuate	***Chitonomyces italicus***
–	Receptacular outgrowth black, straight, with conspicuously hooked apex	***Chitonomyces melanurus***
113	Primary appendage with 1 antheridium, always situated in the lowest cell	***Troglomyces manfrediae***
–	Primary appendage with 3 antheridia situated in the third, fourth and fifth cell	***Troglomyces triandrus***
114	Appendage branched	**115**
–	Appendage an unbranched axis	**116**
115	Basal cell of appendage small, pigmented; appendage cells normal	***Fanniomyces burdigalensis***
–	Basal cell of appendage elongate, not pigmented; appendage cells elongated	***Fanniomyces ceratophorus***
116	Cell VI shorter than cell III; appendage consisting isodiametric to elongated cells	**117**
–	Cells III and VI equally long; appendage with dark basal cell, consisting of flattened cells; on Sphaeroceridae (Diptera)	**120**
117	Venter of perithecium without protuberances	**118**
–	Venter of perithecium with protuberances; on Ephydridae (Diptera)	***Stigmatomyces hydrelliae***
118	Appendage arcuated or sigmoid; perithecial neck shorter than the venter; on *Musca* (Diptera, Muscidae)	***Stigmatomyces baeri***
–	Appendage not arcuated; perithecial neck longer than venter; on *Drosophila* (Diptera, Drosophilidae)	**119**
119	Perithecial neck as long as venter; appendage hyaline, its axis composed of 4 cells; On Drosophila subg. Sophophora (Diptera, Drosophilidae)	***Stigmatomyces majewskii***
–	Perithecial neck 2× as long as venter; appendage brown, its axis composed of 6 cells; On Drosophila subg. Drosophila (Diptera, Drosophilidae)	***Stigmatomyces entomophilus***
120	Venter of perithecium without protuberances	**121**
–	Venter of perithecium with protuberances	**122**
121	Perithecial basal cells elongated, longer than the appendage	***Stigmatomyces limosinae***
–	Perithecial basal cells not elongated, never longer than the appendage	***Stigmatomyces crassicollis***
122	Perithecial apex abruptly becoming conical; appendage not proliferating	***Stigmatomyces platensis***
–	Perithecial apex gradually tapering; appendage proliferating distally	**123**
123	Perithecial venter with numerous knobs, below the neck and also downwards	***Stigmatomyces minilimosinae***
–	Perithecial venter with 4 knobs, only below the neck	***Stigmatomyces divergatus***
124	Basal cells of the primary appendage dark brown on the outer side	***Cryptandromyces euplecti***
–	Primary appendage entirely hyaline	**125**
125	Cell I supporting cell II; thallus > 125 µm long	***Cryptandromyces elegans***
–	Cell I supporting cells II and III; thallus < 100 µm long	***Cryptandromyces bibloplecti***
126	Basal cell of the receptacle with a black upgrowth; on *Rugilus* (Staphylinidae)	***Corethromyces stilici***
–	Basal cell of the receptacle normal, without a black upgrowth; on *Choleva* (Leiodidae)	***Corethromyces henrotii***
127	Upper and lower lobes of perithecium of equal length, not exceeding perithecial tip	***Hesperomyces coccinelloides***
–	Upper lobes long, exceeding the perithecial tip; lower lobes short	**128**
128	On *Halyzia* (Coleoptera, Coccinellidae)	***Hesperomyces halyziae***
–	On *Harmonia*, *Tytthaspis* (Coleoptera, Coccinellidae)	***Hesperomyces virescens* sensu lato**
129	Cell II of receptacle blackened	***Cantharomyces denigratus***
–	Cell II never black, at most brownish or with a black spot	**130**
130	Basal cell of primary appendage supporting a 300–415 µm long unbranched series of 7–11 elongate cells	***Cantharomyces elongatus***
–	Primary appendage not so long	**131**
131	Primary appendage not ramified	***Cantharomyces italicus***
–	Primary appendage with ramifications	**132**
132	Primary appendage ramified above the suprabasal cell; basal cell not spherical	**133**
–	Primary appendage ramified above the basal cell, the latter spherical	***Cantharomyces robustus***
133	Antheridium small, not reaching the upper and lower septa of the basal cell of the appendage	***Cantharomyces platystethi***
–	Antheridium larger, reaching the lower (and upper) septum of the basal cell of the appendage	***Cantharomyces orientalis***
134	Sterile appendages blackish brown, without conspicuous black septa	***Peyritschiella heinemanniana***
–	Sterile appendages hardly pigmented, with black septa	**135**
135	Lower horizontal tier of cells pigmented black, septa between cells obscured	**136**
–	Lower horizontal tier of cells not as pigmented, septa between cells visible	**138**
136	Distal part of receptacle broad; upper tier composed of > 20 cells	***Peyritschiella biformis***
–	Upper tier of the receptacle with < 15 cells; lateral sides of median tier each produce one pigmented outgrowths (some reach the upper tier)	**137**
137	Thallus with 2 perithecia	***Peyritschiella furcifera***
–	Thallus with (2–)3–12 perithecia	***Peyritschiella vulgata***
138	Median cell of both lowest horizontal tiers as long as neighboring cells	***Peyritschiella protea***
–	Median cell of both lowest horizontal tiers much larger than neighboring cells, the latter becoming smaller outwards.	**139**
139	Perithecium usually with auricula; Third and second horizontal tier of similar length	***Peyritschiella dubia***
–	Perithecium without auricula; third horizontal tier longer than second	***Peyritschiella princeps***
140	Secondary receptacula unicellular	**141**
–	Secondary receptacula multicellular	**146**
141	Secondary receptacula pigmented	**145**
–	Secondary receptacula hyaline	**142**
142	Cell VI much longer than perithecium	***Monoicomyces myllaenae***
–	Cell VI shorter than perithecium	**143**
143	Perithecium asymmetrical and bent; antheridium usually without apical branchlets	***Monoicomyces matthiatis***
–	Perithecium symmetrical, straight; antheridium with several apical branchlets	**144**
144	Basal cell of primary appendage at least partially pigmented, not narrowing towards the apex	***Monoicomyces homalotae***
–	Basal cell of primary appendage entirely hyaline, narrowing towards the apex	***Monoicomyces britannicus***
145	Antheridial and primary appendages very long, reaching beyond the perithecial apex	***Monoicomyces fragilis***
–	Antheridial and primary appendages shorter, not reaching beyond the perithecial apex	***Monoicomyces nigrescens***
146	Secondary receptaculum pigmented	***Monoicomyces bolitocharae***
–	Secondary receptaculum hyaline	**147**
147	Secondary and antheridial appendages blackish brown	***Monoicomyces californicus***
–	Secondary and antheridial appendages hyaline or hardly pigmented, never blackish brown	***Monoicomyces invisibilis***


## Supplementary Material

XML Treatment for Hesperomyces halyziae

XML Treatment for Laboulbenia quarantenae
